# Identifying Key Drivers of Efficient B Cell Responses: On the Role of T Help, Antigen-Organization, and Toll-like Receptor Stimulation for Generating a Neutralizing Anti-Dengue Virus Response

**DOI:** 10.3390/vaccines12060661

**Published:** 2024-06-14

**Authors:** Jan M. Sobczak, Irena Barkovska, Ina Balke, Dominik A. Rothen, Mona O. Mohsen, Dace Skrastina, Anete Ogrina, Byron Martina, Juris Jansons, Janis Bogans, Monique Vogel, Martin F. Bachmann, Andris Zeltins

**Affiliations:** 1 Department of Immunology, University Clinic of Rheumatology and Immunology, Inselspital, CH-3010 Bern, Switzerland; dominik.rothen@unibe.ch (D.A.R.); mona.mohsen@unibe.ch (M.O.M.); monique.vogel@unibe.ch (M.V.); martin.bachmann@me.com (M.F.B.); 2 Department of BioMedical Research, University of Bern, CH-3008 Bern, Switzerland; 3 Latvian Biomedical Research and Study Centre, LV-1067 Riga, Latvia; irenabarkovska@gmail.com (I.B.); inab@biomed.lu.lv (I.B.); dace.skrastina@biomed.lu.lv (D.S.); anete.ogrina@biomed.lu.lv (A.O.); jansons@biomed.lu.lv (J.J.); janis.bogans@gmail.com (J.B.); anze@biomed.lu.lv (A.Z.); 4 Artemis Bioservices, 2629 JD Delft, The Netherlands; b.martina@artemisbioservices.com; 5 Protinhi Therapeutics, 6534 AT Nijmegen, The Netherlands; 6 Nuffield Department of Medicine, The Jenner Institute, University of Oxford, Oxford OX3 7BN, UK

**Keywords:** virus-like particles, nanostructures, B-cell responses, dengue virus neutralization

## Abstract

T help (Th), stimulation of toll-like receptors (pathogen-associated molecular patterns, PAMPs), and antigen organization and repetitiveness (pathogen-associated structural patterns, PASPs) were shown numerous times to be important in driving B-cell and antibody responses. In this study, we dissected the individual contributions of these parameters using newly developed “Immune-tag” technology. As model antigens, we used eGFP and the third domain of the dengue virus 1 envelope protein (DV1 EDIII), the major target of virus-neutralizing antibodies. The respective proteins were expressed alone or genetically fused to the N-terminal fragment of the cucumber mosaic virus (CMV) capsid protein—nCMV, rendering the antigens oligomeric. In a step-by-step manner, RNA was attached as a PAMP, and/or a universal Th-cell epitope was genetically added for additional Th. Finally, a PASP was added to the constructs by displaying the antigens highly organized and repetitively on the surface of CMV-derived virus-like particles (CuMV VLPs). Sera from immunized mice demonstrated that each component contributed stepwise to the immunogenicity of both proteins. All components combined in the CuMV VLP platform induced by far the highest antibody responses. In addition, the DV1 EDIII induced high levels of DENV-1-neutralizing antibodies only if displayed on VLPs. Thus, combining multiple cues typically associated with viruses results in optimal antibody responses.

## 1. Introduction

The comprehensive goal for any vaccine candidate is to induce strong and long-lasting antibody (Ab) responses. This can be achieved by displaying the epitopes of interest in a maximally immunogenic manner [1,2,3]. One of the most effective strategies for designing both protective and safe new-generation vaccines involves mimicking the characteristic traits of pathogens, including their size, shape, and surface molecular organization, whilst excluding their infectivity [2,3]. Thus, viral mimetics, such as virus-like particles (VLPs), serve as effective and well-established platforms to endow selected antigens with a viral appearance. VLPs are nanostructures composed of spontaneously assembling proteins that form spherical, rod-shaped, or filamentous particles [4,5,6]. Continuously developed over the past four decades [4,7,8,9,10], VLP-based vaccines have evolved from targeting their source viruses [7,8,11,12,13,14,15,16,17,18,19,20,21,22,23,24,25] to incorporating epitopes from various pathogens, including viruses [26,27,28,29,30,31], bacteria [32,33], and parasites [34,35,36,37,38]. Moreover, they have also been used to address chronic and non-communicable diseases [39,40,41,42,43,44,45,46,47,48]. 

Due to their universal properties, antigen-fused VLPs have attracted significant interest as vaccine vectors, offering a broad spectrum of potential applications [49,50,51,52]. However, fusing an antigen to the viral capsid or coat proteins (CPs)—building blocks of VLPs—may result in undesirable outcomes such as the formation of insoluble inclusion bodies (IBs) [53], alterations in the shape of folded VLPs [5], or even ablated expression. These issues may arise due to the molecular weight of the antigen, its charge, or other sequence peculiarities, including repeats, pseudo-termination sequences, or rare tRNA codons. To address these, various strategies have been developed, including the expression of mosaic VLPs [54,55,56,57,58,59], or the binding of antigens by chemical [39,60,61,62,63,64,65], enzymatic [57,66,67,68,69], or physical [70,71,72,73] methods. 

Beyond the whole viral CPs [50,51,74,75,76,77], the structural elements of CPs, such as the β-annulus peptide [78,79,80], or other genetically encoded proteins capable of self-assembling into multimeric nanostructures, are also potentially applicable in vaccine development. These include ferritin [74,81,82,83,84,85], encapsulin [74,84,86,87,88,89], lumazine synthase [74,84,90,91,92], transferrin [85,93,94,95], lactoferrin [85,96], casein [85,97,98,99], non-viral pyruvate dehydrogenase E2 protein [100,101,102], GCN4-based isoleucine zipper [103,104,105], the T4 bacteriophage fibritin foldon [104,106,107], WA20-foldon (a complex of WA20 protein and T4 bacteriophage fibritin) [108,109], or magnetosomes [85,110,111,112].

Nanostructures, to be used as vaccine platforms, must be able to induce a significant immune response against displayed antigens. Their design should ideally generate robust B- and T-cell responses, including long-lived plasma cells secreting high-affinity Abs [113]. To achieve such favorable immune responses, the dynamics of antigen exposure and innate stimulation must be optimally designed. This can be obtained by modifying both the exterior and interior surfaces of nanostructures, or VLPs [114]. It involves the incorporation of pathogen-associated molecular patterns (PAMPs)—the molecular signatures derived from pathogens and recognized by the immune system—and pathogen-associated structural patterns (PASPs), referring to the spatial arrangement of antigens characteristic of pathogen surfaces [1,2,3]. PASPs are recognized by natural Abs and the complement system, which enhances the uptake of a nanostructure/VLP by an antigen-presenting cell (APC), thereby facilitating T-cell priming [115]. A classic study by Vogelstein and colleagues identified an optimal antigen density on nanoparticles for PASP, which maximizes B-cell activation, to be within the 5–10 nm range [116]. 

In contrast to PASPs, toll-like receptor (TLR)-stimulating PAMP elements included in the interior facets might be as effective as such modifications to the exterior [55,56,59]. The enhancement of immune response was previously demonstrated through the packaging of ssRNA, dsRNA, and CpGs [117,118,119,120], serving as ligands for TLR 7/8, TLR3, and TLR9, respectively [121,122,123,124]. Stimulation of TLR7, for instance, preferentially boosts the production of IgG2a/c and IgG2b subclasses in mice [87,91,125,126], which are crucial for protection against viral [92,127], bacterial [90,128], and parasite infections [93,129].

To enhance Th-cell-dependent B-cell responses, incorporating a strong, universal T-cell epitope can be beneficial [43,59,94]. These can be integrated into the structure of VLP/nanoparticle [43,130], but they are also used as short linear peptides [131,132], fused to proteins [133], coupled to carbohydrates [134], or co-assembled with self-assembling peptide nanofiber systems [135]. Examples of such universal epitopes include a Th-cell epitope from tetanus toxin (TT) [96,136,137,138], the transmembrane domain of the West Nile virus E protein [98,130], the adenovirus Ad5 E1a protein [139], or the synthetic, non-natural Pan DR Epitope (PADRE) [131,140,141].

PADRE is an engineered peptide known for its broad reactivity. It is capable of binding with high or medium affinity to 15 out of the 16 most common human HLA-DR haplotypes [131,141], as well as cross-reacting with mouse class II alleles [140,142]. Thus, PADRE, as a “universal” helper T-cell epitope, has been recognized for its effectiveness in enhancing CD4+ T-cell responses [131,140,141]. Like other T-cell epitopes, it has been utilized as a fusion peptide [131,143,144,145,146,147,148,149,150,151], a carbohydrate carrier [152,153], an adjuvant [143,154,155], and has been incorporated into liposomal formulations [156] or self-assembling nanofibers [157,158,159]. PADRE has been shown to be particularly useful in development vaccine candidates that induce desired self-responses in the context of non-communicable diseases [147,149,154,155,156,158,159], as demonstrated in several pre-clinical trials across various animal models, such as rat arthritis [147] or colorectal cancer [149]. However, its application also extends to the development of distinct protein-based anti-pathogenic vaccines [143,144,145,146,148,150,151,157], including those for *Toxoplasma gondii* [148,150], *Staphylococcus aureus* [157], SARS-CoV-2 [151], or DENV-2 [144]. This demonstrates the versatility of PADRE and suggests the potential benefits of including it in one of the vaccine platforms presented here.

Our research on cucumber mosaic virus (CMV)-derived VLPs, termed “CuMV” in our papers to avoid confusion with cytomegalovirus (which, like cucumber mosaic virus, is abbreviated CMV according to the ICTV classification), demonstrated the spontaneous encapsulation of prokaryotic ssRNA within the particles, which positively correlates with the formation of a high-avidity IgG response [160]. The encapsulation occurs during self-assembly in the bacterial system [55,56,59], a phenomenon consistent with findings regarding Qβ-derived VLPs [161]. Immunological optimization of CuMV VLPs was further carried out by incorporating into their interior facets a universal Th-cell epitope derived from TT (thus forming CuMV_TT_ particles), leading to the development of multiple vaccine candidates that induce highly specific, class-switched neutralizing Abs [43,46,54,55,56,59,60,62,63,65,162,163,164]. However, the limited capacity for antigen fusion in CuMV_TT_ VLPs has prompted us to seek alternative vaccine platforms.

Nearly all plant CPs of icosahedral, positive-sense RNA viruses can be categorized into four distinct structural domains, where the N-terminal “R” domain is involved in the interaction of the viral capsid with genomic RNA [165]. In CMV, the N-terminal fragments of R domains from B and C subunits form a unique bundle of six amphipathic helices oriented down into the virion core case. These helices bind the viral genomic RNA both for its packaging during particle assembly and for maintaining the stability of the assembled particle [166]. This characteristic feature allows the CMV CP N-terminal part (termed here nCMV) to form hexamers post-expression. When fused with a chosen antigen, it may lead to antigen multimerization. Such “immune-tags” [167] may be used as building blocks for vaccine candidates. Furthermore, they provide a novel approach to investigating the individual contributions of Th, PAMPs, and PASPs to the induction of Ab responses.

The use of multimerized N-terminal fragments of CMV CPs containing functional R domains, nCMVs, instead of the entire CuMV_TT_ VLPs offers the versatility of generating multivalent antigens, along with the possibility of attaching TLR-ligands such as RNA. The potential of nCMV as a viable vaccine platform, which we have termed the “Immune-tag” [167], has been investigated here through a mechanistic approach. To this end, the importance of additional Th, PAMPs (TLR7/8 stimulation), and PASPs (antigen multimerization, organization, and repetitiveness) were analyzed both separately and combined. First, the “Immune-tags” carried an enhanced green fluorescent protein (eGFP), serving as a model antigen. eGFP, when used as a “free” antigen, has previously been shown to be poorly immunogenic [168], eliciting only minimal Ab responses, even after the administration of a booster dose [169]. Hence, eGFP may represent a useful protein to demonstrate and quantify the effectiveness of various immunogenicity-enhancing stimuli, including the potential of nCMV-based “Immune-tags”.

To further assess the capability of the “Immune-tag”-based vaccine candidate in a context reflecting authentic medical need, we developed a construct targeting dengue virus 1 (DENV-1). This evaluation aimed to determine its ability to elicit DENV-neutralizing Abs, addressing a significant public health concern in warm-climate regions where *Aedes* mosquito vectors are prevalent [170,171,172]. As an experimental, biologically relevant antigen, we selected the third domain of DENV-1 envelope protein (DV1 EDIII) [173,174,175], which has been identified to contain several serotype-specific neutralizing epitopes [176,177,178,179,180], indicating that anti-EDIII Abs may have reduced potential to mediate the phenomenon of Ab-dependent enhancement (ADE) [180,181,182,183]. In our previous works, we have demonstrated for two other flaviviruses (West Nile and Zika) that the EDIII domain, when expressed alone, folds properly and is capable of inducing neutralizing and protective Abs [60,184].

Our results indicate that antigen multimerization, PAMPs, and extra Th each individually contribute to the elicitation of Ab responses, but the combination of these elements in a non-repetitive antigen carrier does not result in more than an additive increase in response. In contrast, combining all parameters into a highly repetitive, VLP-based vaccine qualitatively enhances the Ab responses. Thus, incorporating PASPs along with extra Th and PAMPs and integrating all stimuli into a single entity was the essential feature for maximal immunogenicity.

## 2. Materials and Methods

### 2.1. Cloning of “Immune-Tag” nCMV-eGFP, nCMV-PADRE-eGFP, nCMV-PADRE-DV1 Variants

**Figure 1:**
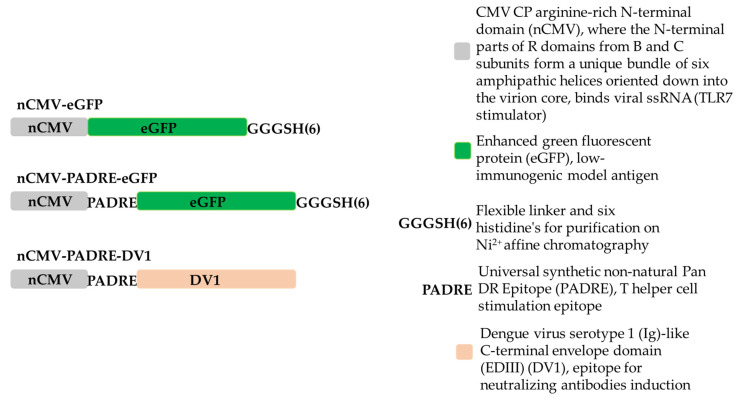
Schematic overview of “Immune-tag” variants used in the study.

To create nCMV-containing constructs, a 171 bp long fragment from the wild-type (WT) CMV *CP* gene with an arginine-rich N-terminal domain (^1^MDKSESTSAGRSRRRRPRRGSRSAPSSADANFRVLSQQLSRLNKTLAAGRPTINHPT^57^), was amplified by PCR using Pfu polymerase (Thermo Fisher Scientific, Waltham, MA, USA) with the oligonucleotides CmN-NcoF and CmN-BamR (Table 1), which contained restriction sites for NcoI (Thermo Fisher Scientific, Waltham, MA, USA) and BamHI (Thermo Fisher Scientific, Waltham, MA, USA), respectively. The pET-CMVwt plasmid [43] was used as the template. The PCR product was extracted from a 0.8% native agarose gel (NAG) using the GeneJet gel extraction kit (Thermo Fisher Scientific, Waltham, MA, USA) following the provided protocol. Subsequently, the PCR fragment was treated with Taq polymerase (Thermo Fisher Scientific, Waltham, MA, USA) to create ddA overlaps for cloning into the ddT of the pTZ57 cloning vector (InsTAclon PCR Cloning Kit, Thermo Fisher Scientific, Waltham, MA, USA). The reaction mixture was prepared according to the protocol, with the addition of 10 mM dATP (Thermo Fisher Scientific, Waltham, MA, USA) instead of the dNTP mix and 10 µL of purified PCR product. The reaction was incubated for 30 min at 72 °C. A linearized pTZ57 ddT vector was then used to clone 1 µL of the reaction mixture, resulting in the creation of the plasmid pTZ-nCMV. The ligates were transformed into XL1-Blue Super competent cells (Agilent Technologies, Santa Clara, CA, USA). Clones containing the insert were verified by Sanger sequencing using the M13seq-F oligonucleotide (Table 1) after test restriction with NcoI and BamHI. The verified pTZ-nCMV clone and a plasmid containing eGFP (pET-eGFP) were cut with HindIII (Thermo Fisher Scientific, Waltham, MA, USA) and BamHI restriction enzymes to clone eGFP at the C-terminus of nCMV, resulting in the development of the pTZ-nCMV-eGFP construct. Subsequently, the nCMV-eGFP and pET-28a(+) (Novagen, Bad Soden, Germany) plasmids were digested with NcoI (partial digestion, as eGFP contains an additional site) and HindIII to develop the pET-nCMV-eGFP expression vector. The constructed vector was selected by digestion with BamHI and HindIII. For rapid purification by Ni^2+^ affinity chromatography, a tag of six histidines (His-tag) was introduced at the C-terminus of eGFP, separated from eGFP by a GGGS flexible linker [185], and the stop codon was removed (note: adding a His-tag at the N-terminal end of nCMV-eGFP reduced expression levels, as indicated by unpublished data). To create this expression vector, two overlapping oligonucleotides, His-tag-C-eGFP-Bsp1407I-F and His-tag-C-eGFP-SacI-R (Table 1), with Bsp1407I and SacI cloning sites (Thermo Fisher Scientific, Waltham, MA, USA), were ordered. Single-stranded oligonucleotide ends were filled by PCR according to the Pfu polymerase protocol, creating a G3S-His-tag PCR product. The PCR product was purified using the QIAquick PCR Purification Kit (Qiagen, Hilden, Germany) according to the provided protocol. Subsequently, pET-nCMV-eGFP and G3S-His-tag were digested with Bsp1704I and SacI. After fragment purification and ligation, the plasmid pET-nCMV-eGFP was created (Figure 1 and Figure S1A). Correct clones were selected by restriction analysis with BamHI and verified by Sanger sequencing using the pET-rev oligonucleotide (Table 1).

To create the PADRE epitope (AKFVAAWTLKAAA [131]) containing construct pET-nCMV-PADRE-eGFP (Figure 1 and Figure S2A), two overlapping oligonucleotides, PADRE-eGFP-BamHI-F and PADRE-eGFP-AgeI-R (Table 1), were used. The same procedure as in the G3S-His-tag case was performed to obtain the PADRE PCR product. Subsequently, pET-nCMV-eGFP and PADRE were digested with AgeI(BshTI) (Thermo Fisher Scientific, Waltham, MA, USA) and BamHI (partial digestion). Fragments were purified as previously described and ligated. Correct clones were selected by restriction analysis with NotI (Thermo Fisher Scientific, Waltham, MA, USA) and verified by Sanger sequencing using the pET-rev oligonucleotide (Table 1).

To create an “Immune-tag” variant with the DENV-1-derived antigen (DV1 EDIII) at the C-terminus of PADRE, replacing eGFP, we developed a universal cloning vector designed for the insertion of antigens with a NdeI restriction site at their 5′ end. Two overlapping oligonucleotides, PADRE-eGFP-BamHI-F and nCMV-Vect_R (Table 1), were utilized in the procedure identical to that used to obtain the PADRE-Nde PCR product. Subsequently, pET-nCMV and PADRE-Nde were digested with AgeI(BshTI) and XhoI (Thermo Fisher Scientific, Waltham, MA, USA). The DV1 coding sequence was excised from the pET-DV1 construct (ordered by gene synthesis, BioCat, Heidelberg, Germany), using the restriction enzymes NdeI and XhoI, (Thermo Fisher Scientific, Waltham, MA, USA) to obtain the pET-nCMV-PADRE-DV1 construct (Figure 1 and Figure S3A).

### 2.2. Protein Production, Purification, and Analysis

The created expression vectors pET-nCMV-eGFP, pET-nCMV-PADRE-eGFP, pET-nCMV-PADRE-DV1 were transformed into the C2566 *Escherichia coli* expression strain (New England Biolabs, Ipswich, MA, USA). Cells were cultivated in kanamycin-supplemented 2TY media (Km; 25 µg/mL) and protein expression was carried out according to the developed protocol used in previous studies [43,57,186]. The biomass collected by low-speed centrifugation (8228× *g*, 5 °C, 5 min) was frozen at −70 °C. Upon thawing on ice, the frozen biomass was suspended in 10 mL of 1× LEW buffer (USB, Cleveland, OH, USA) supplemented with 1 mM phenylmethylsulfonyl fluoride (PMSF; AppliChem, Darmstadt, Germany) and disrupted with ultrasound (Hielscher 200, power 70%, pulse 50%, 16 min) on ice. The solution was clarified by centrifugation (15,557× *g*, 5 °C, 10 min), and the pellet was discarded. The nCMV-eGFP (Figure S1) and nCMV-PADRE-eGFP (Figure S2) proteins were purified using the PrepEase His-Tagged Protein Purification Midi Kit–High Yield (USB, Cleveland, OH, USA) (Figures S1C and S2C). Elution fractions containing nCMV-eGFP and nCMV-PADRE-eGFP were additionally purified by gel-filtration using an Äkta Pure 25 XK 16/70 column packed with 120 mL Superdex™ 200 (Cytiva, Marlborough, MA USA) (Figures S1D,E and S2D,E). Purified proteins were eluted with PBS at a flow rate of 1 mL/min, collecting 2 mL per fraction. Following analysis by sodium dodecyl sulfate-polyacrylamide gel electrophoresis (SDS-PAGE), 1 mM PMSF was added to the gel-filtrated fractions. The fractions containing nCMV-eGFP and nCMV-PADRE-eGFP were pooled and concentrated using Amicon^®^ Ultra-15, 10 KDa MWCO filtration units (Merck-Millipore, Darmstadt, Germany). The samples were then fresh-frozen and stored at −70 °C.

The cells containing nCMV-PADRE-DV1 (Figure S3) were resuspended in 10 mL of PBS and disrupted as described above. After centrifugation (15,557× *g*, 5 °C, 10 min), the supernatant was discarded. nCMV-PADRE-DV1 was purified from IB using a single freeze-thawing cycle method [187]. Briefly, pellets containing IB were resuspended in 10 mL of wash buffer (50 mM Tris-HCl pH 8.0, 100 mM NaCl, 1 mM EDTA, 1% TX-100, 1 M urea) by ultrasound (Hielscher 200, power 70%, pulse 50%, 5 min) on ice. IB were collected by centrifugation (15,557× *g*, 5 °C, 10 min), and the supernatant was discarded. IB washing was repeated two more times. To remove residues of TX-100, IB were washed with PBS. Subsequently, IB were resuspended in solubilization/refolding buffer 100 mM CAPS pH 9.5, arginine 0.9 M, 0.3 mM reduced glutathione (GSH), 0.03 mM oxidized glutathione (GSSG), and 3 M urea and frozen at −20 °C for 16 h. The refolding reaction mixture contains the chaotropic agent, urea, which provides efficient removal of bound impurities from the proteins [188]. Refolded nCMV-PADRE-DV1 was thawed for 1 h at RT on a rotator set at 10 rpm. The supernatant was clarified by centrifugation (15,557× *g*, 5 °C, 10 min), and the pellet was discarded. nCMV-PADRE-DV1 was dialyzed against 20 mM Na phosphate buffer, pH 9.0, in a 6–8 kDa dialysis membrane (Spectrum Laboratories, Rancho Dominguez, CA, USA). Additionally, nCMV-PADRE-DV1 was purified by gel-filtration on an Äkta Pure 25 XK 16/70 column packed with 120 mL Superdex™ 200, and purified proteins were eluted with PBS at a flow rate of 1 mL/min, collecting 2 mL per fraction (Figure S3D,E). After analysis by SDS-PAGE, nCMV-PADRE-DV1-containing fractions were pooled and concentrated using Amicon^®^ Ultra-15, 10 KDa MWCO filtration units (Merck-Millipore, Darmstadt, Germany).

eGFP for chemical conjugation was expressed from pQE-eGFP (kindly provided by SIA Asla Ltd.), which was transformed into the *E. coli* C2566 strain. Expression was performed as described above, with minor modifications in the expression step. Cells were grown at 37 °C until an OD600 of 0.8 was reached, then supplemented with 5 mM MgCl_2_ and induced with 0.2 mM isopropyl-β-D-thiogalactoside (IPTG; Thermo Fisher Scientific, Waltham, MA, USA), and cultivated overnight (ON) at 37 °C and 200 rpm/min (7 × g). The pellet was resuspended in 10 mL of 1× LEW buffer and disrupted with ultrasound (Hielscher 200, power 70%, pulse 50%, 16 min) on ice. The subsequent purification steps were the same as described for nCMV-eGFP and nCMV-PADRE-eGFP (Figure S4).

DV1 EDIII (MW 13.694 kDa) (Figure S5) was purified from IB by a single freeze-thawing cycle method [187] (Figure S5C), as described for nCMV-PADRE-DV1. Instead of the Superdex™ 200 gel-filtration column, Superdex 75™ (Cytiva, Marlborough, MA USA) was used for purification (Figure S5D,E).

All obtained proteins were soluble post-refolding and purification, which supports their structural integrity and native conformation, as proteins lacking correct folding tend to form aggregates [189]. They were subsequently analyzed by 12.5% SDS-PAGE, 0.8% NAG, mass spectrometry (MS), and dynamic light scattering (DLS). Protein concentration was determined using the Qubit 2.0 (Thermo Fisher Scientific, Waltham, MA, USA) with the Qubit™ Protein Assay Kit (Thermo Fisher Scientific, Waltham, MA, USA) following the manufacturer’s protocol. All buffers used for the final preparation of protein samples were prepared under sterile conditions, utilizing autoclaved Milli-Q water, and filtered through a 0.22 µm filter.

### 2.3. Expression and Purification of CuMV and CuMV_TT_ VLPs

The expression and purification of WT CuMV VLPs and “immunologically optimized” CuMV_TT_ VLPs were performed according to the protocols outlined by Zeltins et al. [43], Storni et al. [46], and Sobczak et al. [59]. In summary, RNA was extracted from CMV-infected lily leaves and reverse-transcribed to cDNA. The resultant PCR products were then inserted into the pTZ57R/T vector (Fermentas, Vilnius, Lithuania). Following sequencing, the CMV *CP* gene was transferred into the pET28a(+) vector using NcoI and HindIII restriction sites. For CuMV_TT_, a TT epitope was integrated into the CMV *CP* gene via a two-step PCR-based mutagenesis approach. For protein expression, *E. coli* C2566 cells were transformed with the pET-CuMVwt or pET-CuMV_TT_ plasmid harboring the CuMV or CuMV_TT_ *CP* gene. Collected cell biomass was pelleted by centrifugation (2600× *g*, 4 °C, 10 min), resuspended in lysis buffer, and disrupted using a sonicator (Hielscher UP200S, Amplitude 70%, cycle 0.5) for 16 min. Post-sonication, the lysate was centrifuged (15,000× *g*, 4 °C, 10 min), and self-assembled VLPs were precipitated from the soluble fraction by adding 3 M ammonium sulfate, followed by ON incubation at 4 °C. After subsequent centrifugation (15,000× *g*, 4 °C, 10 min), the pellet was dissolved in sodium borate buffer (5 mM borate, 2 mM EDTA; pH 9.0) and subjected to ultracentrifugation through a 20–60% sucrose gradient (110,000× *g*, 18 °C, 6 h) using a SW32 Ti rotor (Beckman Coulter, Brea, CA, USA). Fractions containing VLPs were further purified twice by ultracentrifugation through a 30% sucrose cushion (250,000× *g*, 4 °C, 4 h) using a Type 70 Ti rotor (Beckman Coulter, Brea, CA, USA) for LPS removal. The pellet obtained after the first ultracentrifugation was dissolved in sodium borate buffer, and the pellet obtained after the second ultracentrifugation was dissolved in VLP storage buffer (5 mM NaP, 2 mM EDTA; pH 7.5) and stored at 4 °C. Quality control was conducted via SDS-PAGE, NAG, and transmission electron microscopy (TEM). The colorimetric Pierce™ BCA Protein Assay (Thermo Fisher Scientific, Waltham, MA, cat. 23227) was used for the determination of total protein concentration in a final VLP sample. Purified VLPs were tested for endotoxin content, showing less than 100 EU/mg of protein as measured by the Limulus Amebocyte Lysate (LAL) assay (Pierce LAL Chromogenic Endotoxin Quantitation Kit, Thermo Fisher Scientific, Waltham, MA, cat. 88282) [190]. This value is significantly below the endotoxin limits typically set for pharmaceuticals used in mouse models during preclinical research [191,192]. All buffers used for the final preparation of CuMV and CuMV_TT_ VLPs were prepared under sterile conditions, utilizing autoclaved Milli-Q water, and filtered through a 0.22 µm filter.

### 2.4. Isolation and Quantification of RNA from CuMV Samples

To quantify the RNA content of the VLPs, 250 µL of TRIzol reagent was added to 200 µg of CuMV VLPs and mixed well [193]. The sample was then incubated on ice for 10 min and centrifuged (12,000× *g*, 4 °C, 10 min). The supernatant was transferred to a new tube, and the pellet was discarded. 50 µL of precooled chloroform was added, followed by vortexing for 15 s. The mixture was then incubated on ice for additional 10 min and centrifuged (12,000× *g*, 4 °C, 15 min). The upper aqueous phase was gently transferred to a new tube, avoiding the lower organic phase. Next, 125 µL of ice-cold isopropanol was added to precipitate the RNA. Tubes were inverted 10 times (avoiding vortexing) to mix the contents, followed by a 10 min incubation on ice. The mixture was centrifuged (12,000× *g*, 4 °C, 10 min), and the supernatant was discarded. The RNA pellet was washed with 150 µL of 75% ethanol and gently vortexed. Next, brief centrifugation (8000× *g*, 4 °C, 5 min) was performed to re-pellet the RNA. The supernatant was discarded, and the RNA pellet was dried on a heat block set to 24 °C for 10 min and then resuspended in 100 µL of DEPC-treated water. The pellet was dissolved by gentle pipetting and incubated at 55 °C for 10 min to ensure complete solubilization. The final RNA concentration was determined using a NanoDrop spectrophotometer, showing a yield of 214.5 ng/µL from the initial 200 µg of CuMV, establishing a ratio of roughly 10:1 protein-to-RNA.

### 2.5. CMV CP mRNA Transcription

To obtain WT CMV *CP* mRNA, 10 µg of the WT CMV *CP*-containing plasmid under the *T7* polymerase promoter created by Zeltins and co-workers [43] was linearized using the HindIII restriction enzyme for 3 h at 37 °C. The linearized plasmid was then purified with a GeneJet gel extraction kit according to the provided protocol, and its concentration was measured using a NanoDrop-1000 spectrophotometer. Subsequently, 1 µg of linearized plasmid was used for WT CMV *CP* mRNA transcription following the TranscriptAid T7 High Yield Transcription kit (Thermo Fisher Scientific, Waltham, MA, USA) manual. Synthesized *CP* mRNA was purified using a GeneJet RNA purification kit (Thermo Fisher Scientific, Waltham, MA, USA) as per the manufacturer’s protocol and analyzed on a 1% NAG. The concentration of WT CMV *CP* mRNA was determined using a NanoDrop-1000 spectrophotometer.

### 2.6. nCMV Binding to Nucleic Acid

To test whether the nCMV is able to bind the nucleic acid, 1 μg of purified WT CMV CP mRNA was incubated with 1 μg of either eGFP, serving as the negative control, or the nCMV-eGFP vaccine [194] for 10 min on ice in their respective storage buffers. Samples were treated with Benzonase^®^ (25 units/μL; Novagen, Bad Soden, Germany) to degrade unbound RNA in the solution. The nucleoprotein complex was analyzed using 0.8% NAG stained with ethidium bromide (Figure S6). Furthermore, the ability of nCMV-PADRE-DV1 to bind the WT CMV *CP* mRNA has been tested with a ratio of 6:1 protein-to-RNA (Figure S3F,G). The 6:1 protein-to-RNA ratio was established to match the equivalent VLP protein-to-RNA ratio, accounting for the potential loss of a small fraction of RNA during isolation from VLPs [195]. This ratio also considers the unknown efficiency of RNA binding to nCMV “Immune-tags”, and is based on the fact that six N-terminal R domains of the CMV CP form a helical bundle upon interacting with RNA.

### 2.7. Electrophoretic Nucleic Acid Mobility Shift Assay (Gel Shift)

To evaluate the binding capacity of “Immune-tags” to nucleic acids, seven samples of each of the three vaccine variants—eGFP, nCMV-eGFP, and nCMV-PADRE-eGFP—were prepared at seven distinct doses: 0 ng, 150 ng, 300 ng, 600 ng, 800 ng, 1200 ng, and 1500 ng. A uniform concentration of 1 μg per sample of WT CMV *CP* mRNA was introduced to each sample to reach a final reaction volume of 20 µL. Following the addition of the nucleic acid, the samples were chilled on ice for 10 min before being subjected to analysis using a 1% NAG gel. Furthermore, the nCMV-eGFP vaccine variant was tested for its ability to bind to other types of nucleic acids: the CMV CP PCR product, the ryegrass mottle virus (RGMoV) *CP* mRNA, and the Type A CpG TLR9 agonist, G10.

### 2.8. Development of CuMV-eGFP and CuMV_TT_-DV1 Vaccines

Before chemical conjugation, eGFP lysins were modified with the chemical linker N-succinimidyl S-acetyl thioacetate (SATA; Thermo Fisher Scientific, Waltham, MA, USA) at a 10-molar excess to eGFP following the provided manufacturer protocol. Unreacted SATA and deacetylation solutions were removed using Amicon^®^ Ultra-0.5, 10 KDa MWCO filtration units (Merck Millipore, Darmstadt, Germany). The chemical conjugation of eGFP to WT CuMV VLPs was achieved using the cross-linker succinimidyl 6-((β-maleimidopropionamido)hexanoate)—SMPH (Thermo Fisher Scientific, Waltham, MA, USA; approved for usage in clinical trials [196,197,198]) at 10-molar excess to WT CMV CP for 1 h at RT. The chemical conjugation reaction was performed by shaking (1400 rpm, RT, 3 h) on a DSG Titertek shaker (Flow Laboratories, Oldham, UK) with a molar ratio of eGFP/CMV CP (1:1). Unreacted SMPH and uncoupled eGFP were removed using Amicon^®^ Ultra-0.5, 100 KDa MWCO filtration units (Merck Millipore, Darmstadt, Germany).

For DV1 chemical conjugation to CuMV_TT_ VLPs, the cross-linker SMPH was used at five molar excesses to CMV CP for 1 h at RT. The chemical conjugation reaction was performed as described above. Unreacted SMPH was removed using Amicon^®^ Ultra-0.5, 100~KDa MWCO (Millipore, Billerica, MA, USA) filtration units. The uncoupled DV1 was removed by gel-filtration on Superdex™ 200 (Cytiva, Marlborough, MA, USA). Concentrations of CuMV-eGFP and CuMV_TT_-DV1 were measured on the ND-1000 and using the Qubit 2.0 (Thermo Fisher Scientific, Waltham, MA, USA). Coupling efficiency was calculated by gel densitometry analysis [43], resulting in approximately 23% for CuMV-eGFP and 17% for CuMV_TT_-DV1 efficiency. Samples were analyzed by SDS-PAGE, NAG, TEM, and DLS.

### 2.9. Dynamic Light Scattering Measurement (DLS)

VLPs and “Immune-tags” at concentrations of 0.5–1 mg/mL were analyzed in a low-volume glass cuvette (12 µL) using a Zetasizer Nano ZS instrument (Malvern Instruments Ltd., Malvern, UK). The average hydrodynamic diameter of VLPs, or “Immune-tags”, was calculated from three consecutive measurements. Results were analyzed by Zetasizer software (version 8.01, Malvern Instruments Ltd., Malvern, UK).

### 2.10. Sample Analysis by Mass Spectrometry (MS)

Samples for MS analysis were prepared as follows: 2 µL of purified protein (0.5–1 mg/mL) was mixed with 2 µL of 2% trifluoroacetic acid and 2 µL 2,5-dihydroxyacetophenone (2,5-DHAP; Bruker Daltonics, Leipzig, Germany) matrix solution (50 μM 2,5-DHAP dissolved in 96% ethanol and 10 μM aqueous diammonium hydrogen citrate). This mixture was subsequently applied in a volume of 1 µL onto an MTP Anchor Chip 400/384TF (Bruker Daltonics, Leipzig, Germany) and left to crystallize. The sample analysis was performed using an AutoFlex MALDI-TOF MS (Bruker Daltonics, Leipzig, Germany), with mass calibration standard I (Bruker Daltonics, Leipzig, Germany) used for mass calibration.

### 2.11. Transmission Electron Microscopy (TEM)

5 µL of the sample (1 mg/mL) were adsorbed onto carbon formvar-coated copper grids for 3 min. The grids were then drained by taping one grid edge to the filter paper and washed with 1 mM EDTA. Subsequently, the grids were negatively stained with 5 µL of 0.5% uranyl acetate aqueous solution for 1 min. After staining, the excess staining solution was removed by taping one grid edge to the filter paper and draining in 1 mM EDTA. The grids were examined using a JEM-1230 TEM (JEOL, Tokyo, Japan) at an accelerating voltage of 100 kV or 80 kV.

### 2.12. Mice

nCMV-eGFP, nCMV-PADRE-eGFP, and CuMV-eGFP immunization experiments were performed using (8–12 weeks old) WT female BALB/c mice purchased from the Laboratory Animal Center at the University of Tartu (Laboratory Animal Center, Tartu, Estonia). All animals were treated for experimentation according to protocols approved by the Animal Protection Ethics Committee of the Latvian Food and Veterinary Service of the Republic of Latvia, permission No. 89, and were conducted in compliance with Directive 2010/63/EU as adopted by the national legislation.

nCMV-PADRE-DV1, CuMV_TT_-DV1, and DV1 EDIII immunization experiments were performed using (8–12 weeks old) WT female BALB/cOlaHsd mice purchased from Envigo (Envigo Rms B.V., Horst, The Netherlands). All experiments were conducted according to protocols approved by the Swiss Cantonal Veterinary Office (license no. BE 70/18).

### 2.13. Immunization Regimen

**Figure 2:**
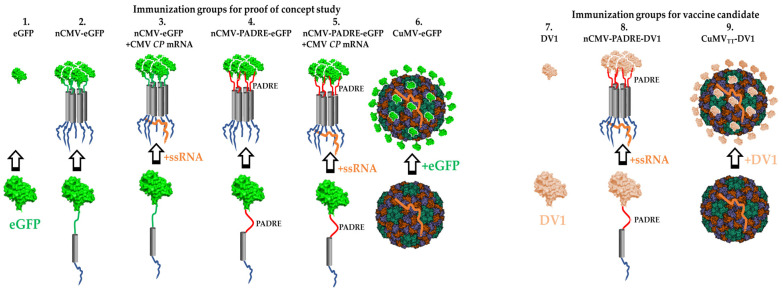
Schematic overview of immunization groups. (**1**) control group immunized with plain eGFP; (**2**) immunized with nCMV-eGFP; (**3**) immunized with nCMV-eGFP supplemented with CMV *CP* mRNA (ssRNA for TLR7 activation) in a protein-to-RNA ratio 6:1; (**4**) immunized with nCMV-PADRE-eGFP; (**5**) immunized with nCMV-PADRE-eGFP supplemented with CMV *CP* mRNA (ssRNA for TLR7 activation) in a protein-to-RNA ratio 6:1; (**6**) immunized with chemically conjugated variant CuMV-eGFP; (**7**) control group immunized with plain DV1; (**8**) immunized with nCMV-PADRE-DV1 supplemented with CMV *CP* mRNA (ssRNA for TLR7 activation) in a protein-to-RNA ratio 6:1; (**9**) immunized with chemically conjugated variant CuMV_TT_-DV1.

The immunogenicity of the created nCMV-eGFP and nCMV-PADRE-eGFP “Immune-tags”, as well as their components, in comparison to CuMV-eGFP and “free” eGFP, was tested in mice through subcutaneous (s.c.) injections with a dose of 50 µg diluted in 1× PBS (without adjuvants) up to a total volume of 200 µL. The injection dose was selected based on our previous immunization study with non-adjuvanted VLPs [57] and the reported low immunogenicity of eGFP [168,169]. The mice were divided into six groups (Figure 2), each consisting of five mice: control group (1) received plain eGFP protein, while the experimental groups (2–6) received injections with the following constructs: nCMV-eGFP (2), nCMV-eGFP supplemented with WT CMV *CP* mRNA in a ratio of 6:1 (3), nCMV-PADRE-eGFP (4), nCMV-PADRE-eGFP supplemented with WT CMV *CP* mRNA in a ratio of 6:1 (5), and CuMV-eGFP (6). Booster injections were administered with the same dose for each variant on days 14 and 28. The experiment was terminated on day 42.

The immunogenicity of the created nCMV-PADRE-DV1 “Immune-tag” (supplemented with CMV *CP* mRNA in a 6:1 protein-to-RNA ratio) in comparison to CuMV_TT_-DV1 and “free” DV1 EDIII (Figure 2) was tested in mice by s.c. injections with a dose of either 30 or 10 µg diluted in PBS up to a total volume of 200 µL. These doses were selected based on our previous dose escalation study with a CuMV_TT_-based vaccine [59]. The higher dose (30 µg) aimed to estimate the immunogenicity of developed vaccines, and the lower dose (10 µg) aimed to explore the efficacy of vaccines in inducing a protective immune response measured by an *in vitro* focus reduction neutralization test. Booster injections were performed with the same dose for each variant on day 14. The experiments were terminated on day 35.

The doses of all vaccines were calculated based on the protein content of the active substance. Quantitative analysis of the protein content for each vaccine was performed to ensure accurate dosing and to evaluate the consistency of vaccine formulations. All immunizations were performed with sterile vaccines that were filtered through a 0.22 µm filter.

### 2.14. The Enzyme-Linked Immunosorbent Assay (ELISA)

The total IgG Abs titers in the sera of immunized mice against eGFP and WT CMV CP were measured in 96-well ELISA plates (Nunc Immuno MaxiSorp, Rochester, NY, USA, Thermo Fisher Scientific, Waltham, MA, USA) coated either with eGFP or CuMV VLPs diluted in 50 mM sodium carbonate buffer, pH 9.6, at a concentration of 10 µg/mL (100 µL per well), and incubated at 4 °C ON. Plates were washed three times with a washing solution (PBS, 0.05% Tween-20) and then rinsed with dH_2_O. Subsequently, plates were blocked with 1% BSA in PBS (100 µL per well) at 37 °C for 30 min, followed by washing as previously described. Mouse sera were added to the plates in PBS containing 1% BSA, starting at a dilution of either 1:50 or 1:400. Specifically, a 1:50 pre-dilution was used for sera from mice immunized with either eGFP or "Immune-tag" constructs added to plates coated with either eGFP or CuMV VLPs. A 1:50 pre-dilution was also used for sera from mice immunized with CuMV-eGFP added to plates coated with CuMV VLPs. A 1:400 pre-dilution was used for sera from mice immunized with CuMV-eGFP added to plates coated with eGFP. Serial dilutions of pre-diluted sera were performed with a dilution ratio of 1:2. Plates were incubated at 37 °C for 1 h, followed by washing as before. Rabbit anti-mouse IgG, conjugated with horseradish peroxidase (HRP) (Sigma–Aldrich, St. Louis, MO, USA, cat. A9044-2ML), was added at a dilution of 1:10,000 (100 µL per well). Plates were incubated at 37 °C for 1 h and washed as previously described. The OPD substrate tablet (o-phenylenediamine dihydrochloride; Sigma–Aldrich, St. Louis, MO, USA) was dissolved in 10 mL of 50 mM sodium carbonate buffer, pH 9.6, with the addition of 15 µL of H_2_O_2_. Then, 100 µL of substrate solution was added to each well, and plates were incubated at 37 °C for 20 min. The reaction was terminated by adding 1.2 N H_2_SO_4_ in a volume of 50 µL per well. Optical absorbance was measured at 492 nm (OD492) using a Labsystems Multiskan MS Type 352 microplate reader (Labsystems Diagnostics Oy, Vantaa, Finland). The endpoint titers were calculated as the highest serum dilution that resulted in an absorbance value exceeding three times that of the negative control (serum obtained from non-immunized mice) [199,200].

Isotype-specific ELISA was performed using the mouse monoclonal Ab isotyping reagent ISO2 (Sigma–Aldrich, St. Louis, MO, USA, cat. ISO2-1KT) and a secondary anti-goat/sheep IgG, HRP Abs (Sigma–Aldrich, St. Louis, MO, USA, cat. A9452-1VL). For the determination of IgG1 and IgG2a subclass levels in the sera of mice immunized with constructs containing eGFP, the following Abs were used in ELISA: goat anti-mouse IgG1, HRP (1:1000) (Thermo Fisher Scientific, Waltham, MA, USA, cat. PA1-74421) and goat anti-mouse IgG2a, HRP (1:1000) (Thermo Fisher Scientific, Waltham, MA, USA, cat. A-10685). Endpoint titers were calculated as described previously.

Total IgG Abs titers in sera of immunized mice against DV1 EDIII were measured in 96-well half-area ELISA plates (Corning Inc., Corning, NY, USA) coated with DV1 EDIII diluted in 1× PBS with a concentration of 2 µg/mL (50 µL per well), incubated at 4 °C ON. On the following day, plates were washed five times with PBS and blocked with 0.15% casein in PBS (100 µL per well) at RT for 2 h followed by washing as previously described. Mouse sera were added to the plates in PBS containing 0.15% Casein, starting at a dilution of either 1:30 or 1:10. Specifically, a 1:30 pre-dilution was used for sera from mice immunized with 30 µg of either DV1 EDIII, nCMV-PADRE-DV1, or CuMV_TT_-DV1. A 1:10 pre-dilution was used for sera from mice immunized with 10 µg of either nCMV-PADRE-DV1 or CuMV_TT_-DV1. Serial dilutions of the pre-diluted sera were then performed with a dilution ratio of either 1:5 or 1:3, respectively. Plates were incubated at RT for 1.5 h and washed as previously. Subsequently, a goat anti-mouse IgG conjugated to HRP (Jackson ImmunoResearch, West Grove, PA, USA; cat. 115 035 008) was added with a dilution factor of 1:5000 and incubated at RT for 1 h. Following incubation, plates were washed five times with PBS-Tween 0.01%. The ELISA was developed with a 50 µL solution of tetramethylbenzidine (TMB), H_2_O_2,_ and acetate buffer (pH 4.1). The reaction was terminated after 5 min by the addition of 50 µL of 1 M H_2_SO_4_ per well. Optical absorbance was measured at an OD of 450 nm (OD450) on an ELISA plate reader. Half-maximal Ab titers (OD_50_) were defined as the reciprocal of the dilution leading to half of the OD measured at saturation.

The avidity of vaccine-induced DV1 EDIII-specific IgG was tested through the avidity ELISA assay performed by extending the ELISA protocol with additional washing steps with a chaotropic agent---urea [55,56,164,201]. Following serum incubation with a pre-dilution of 1:20 and serial dilution of 1:3, the plates were washed with PBS containing 0.01% Tween. The plates were then incubated three times with 7 M urea in PBS containing 0.01% Tween for 5 min on a shaker at RT, or with PBS containing 0.01% Tween as a control. Between these incubations, plates were washed again with PBS-Tween 0.01%. The avidity index (AI) was calculated by the ratio of dilution factors (titers) with and without urea denaturation [202,203,204].

### 2.15. DENV-1 Focus Reduction Virus Neutralization Test (FRNT)

The DENV-1 neutralization capacity of antibodies produced after immunizing mice was determined using focus reduction virus neutralization test (FRNT). Human convalescent serum 001 to Dengue Virus (NR-50226) was used as positive control, while sera from naïve mice were used as a negative control. Briefly, a day before the experiment 96-well substrate plate was prepared using Vero cells (2 × 10^4^ cells/well) cultured in complete media. The sera from vaccinated mice were heat-inactivated (HI) at 56 °C for 30 min to inactivate complement and diluted 1:100 in infection media (composition: DMEM supplemented with 0.75% NaHCO_3_, 10 mM HEPES buffer, 1% Pen-Strep, and 1% HI-FBS). The 500 TCID50 of DENV-1 (VR1856, Hawaii) was added to each well in an equal volume and the mixture was incubated at 37 °C for 1 h. The mixtures of virus and sera were then added to the substrate plate and incubated for 1 h at 37 °C in 5% CO_2_. Subsequently, the cell monolayers were washed once with PBS followed by the addition of the infection media and incubated at 37 °C in 5% CO_2_ for another 48 h. To determine the number of virus-infected cells an in-house developed immunostaining method was used. Briefly, the infected cells were fixed with 2.5% formalin and permeabilized with 0.1% Triton X-100 in 70% ethanol. The infected cells were stained with Rabbit anti-flavivirus group antigen monoclonal antibody (Absolute antibody, Oxford, UK) and detected with Goat anti-rabbit IgG (H+L) Highly Cross-Adsorbed Secondary Antibody, Alexa Fluor Plus 488 (2 mg), (Invitrogen, Waltham, MA, USA). Following this, wells were incubated with 4′,6-Diamidino-2-Phenylindole, Dihydrochloride (DAPI) (Thermo Fisher Scientific, Waltham, MA, USA) to counterstain the nucleus. Plates were scanned using Cytation 1 Imaging Reader (BioTek, Winooski, VT, USA) at a 4× objective and analysed by the Gen5 software (BioTek, Winooski, VT, USA). The percentage reduction of DENV-1-infected cells was determined by comparing the number of infected cells to the negative control. Each serum sample was tested in duplicate.

### 2.16. Data Analysis

Statistical analysis of the collected data was conducted using GraphPad Prism Version 8 (Graph Pad Software Inc., San Diego, CA, USA). For comparisons among groups exhibiting a normal distribution, Student’s *t*-tests or ANOVA were used, incorporating Welch’s correction for the assumptions of unequal variances across groups. For groups not following a normal distribution, the Kruskal-Wallis nonparametric test was used. Differences between groups with *p*-values of 0.05 or less were considered statistically significant (* *p* ≤ 0.05, ** *p* ≤0.01, *** *p* ≤ 0.001, and **** *p* ≤ 0.0001).

## 3. Results

### 3.1. Development of “IMMUNE-TAG” as a Vaccine Platform

**Figure 3:**
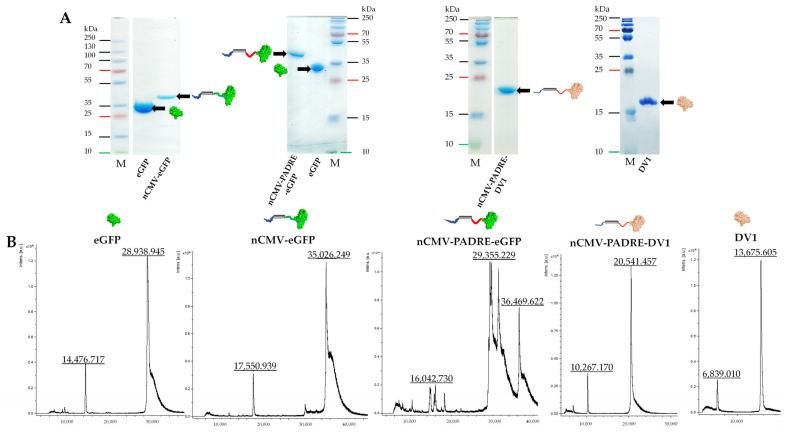
Molecular mass and integrity analysis of developed “Immune-tags”. (**A**)—SDS-PAGE stained with Coomassie G250 stain; M—protein molecular weight marker (PageRuler^TM^ Plus, Thermo Fisher Scientific, Waltham, MA, USA, cat. 26619); eGFP—enhanced green fluorescent protein (MW 29 kDa); nCMV-eGFP (MW 35 kDa); nCMV-PADRE-eGFP (MW 36.5 kDa); nCMV-PADRE-DV1 (MW 20.5 kDa); DV1—immunoglobulin (Ig)-like C-terminal domain of the DENV-1 envelope protein (EDIII) (MW 13.7 kDa); (**B**)—mass spectrometric analysis of the purified “free” proteins and “Immune-tag” constructs.

To dissect the roles of PAMPs, PASPs, and Th stimulation in driving B cell responses, we designed the nCMV as a novel vaccine platform, the “Immune-tag” [167]. Our investigation focused on its ability to oligomerize post-purification and to elicit Ab responses against model antigens, enhanced by the addition of TLR and T-cell-stimulating epitopes. The “Immune-tag” design facilitates swift antigen replacement, cost-effective purification, and the incorporation of immunostimulating components. To begin with, we developed two “Immune-tag” constructs to examine their feasibility. In the first construct, the C-terminal part of nCMV was genetically fused to eGFP (nCMV-eGFP; Figure 1, Figure 2 and Figure S1), while the second incorporated an additional PADRE epitope between nCMV and eGFP (nCMV-PADRE-eGFP; Figure 1, Figure 2 and Figure S2). Both “Immune-tag” variants were expressed in *E. coli* at high concentrations and in soluble form (Figures S1C and S2C). “Immune-tags” and a “free” eGFP serving as a control were analyzed on SDS-PAGE (Figure 3A) and by MS (Figure 3B) after purification and concentration. During MS analysis, partial proteolysis of nCMV-PADRE-eGFP was observed, revealing two peaks: one corresponding to the full length of nCMV-PADRE-eGFP (36.47 kDa) and the second to the eGFP (29.36 kDa) (Figure 3B). The addition of protease inhibitors such as PMSF was sufficient to protect the construct against proteolysis after the gel-filtration step (Figure 3A and Figure S2D), which allowed for the separation of the intact nCMV-PADRE-eGFP from a processed part. On the other hand, the MS analysis demonstrated the integrity of the nCMV-eGFP “Immune-tag” with a corresponding size of 35.03 kDa (Figure 3B).

To assess the capacity of nCMV for nucleic acid binding, gel shift assays were conducted. The assays revealed a migration delay in WT CMV *CP* mRNA in NAG, indicating the retained ability of nCMV to bind mRNA encoding WT CMV CP (Figures S1F and S2F). Considering that the binding of nucleic acids by nCMV relies on electrostatic interactions, these results suggested that nCMV had the potential to bind nucleic acids from various sources. To examine this feature of nCMV, we tested the effect of supplementing nCMV-eGFP with WT CMV *CP* PCR product, as well as RGMoV *CP* mRNA [205], and with Type A CpG TLR9 agonist—G10 [206,207], observing the same migration delay of the construct as for WT CMV *CP* mRNA (Figure S7). Further analysis of the nCMV-eGFP and nCMV-PADRE-eGFP constructs, both with and without RNA, was performed using DLS and TEM. These techniques verified the oligomer formation (Figures S1G–J and S2G–J), with the prospective ability to facilitate augmented Ab responses against target antigens. Both “Immune-tag” variants, enriched with WT CMV *CP* mRNA, were used in subsequent mouse immunization experiments to examine whether nCMV oligomerization and supplementation with TLR7/8 ligand could serve as self-adjuvants.

To employ another antigen, in exchange to the model eGFP antigen (Section 3.3, Section 3.4 and Section 3.5), the “Immune-tag” was adapted for DENV-1. In this adaptation, the DV1 EDIII sequence derived from DENV-1 (Figure S5A) was integrated into the platform by replacing eGFP, thus creating a new construct, nCMV-PADRE-DV1 (Figure S3B). Both the nCMV-PADRE-DV1 “Immune-tag” (Figure S3) and “free” DV1 EDIII (Figure S5) following purification were analyzed on SDS-PAGE and by MS (Figure 3), to confirm the integrity and expected molecular properties of the nCMV-PADRE-DV1 construct. Before immunization, nCMV-PADRE-DV1 was supplemented with CMV *CP* mRNA in a protein-to-RNA ratio of 6:1 (Figure S3F,G).

### 3.2. Chemically Modified VLP-Based Platform

The comparative analysis aimed to understand the impact of antigen spatial organization and repetitiveness (PASP) on the efficiency of Ab induction and was conducted utilizing a selected model antigen—eGFP. eGFP was either genetically fused with the “Immune-tags” or chemically coupled to the surface of structurally uniform VLPs (Figure 2). To compare their immunological potentials without additional confounding variables, eGFP was conjugated to VLPs derived from WT CMV (CuMV), lacking integration with TT epitopes. This design avoids direct comparison between the PADRE and the TT CD4+ epitopes, thereby allowing a clearer assessment of the influence of antigen organization and repetitiveness against other B-cell stimulatory features incorporated into the “Immune-tag” constructs.

In the second phase of the project, when focusing principally on identifying a potent vaccine candidate against DENV-1, DV1 EDIII antigen was conjugated with “immunologically optimized” VLPs (CuMV_TT_), which do incorporate a universal T-cell epitope from TT. This fusion aims to boost the interaction between Th cells and B cells and has been shown to significantly enhance Ab responses in mice that were primed with TT before being immunized with CuMV_TT_ [59,160]. In essence, the incorporation of the TT epitope is designed to leverage the widespread pre-existing immunity to TT within the human population, potentially leading to improved vaccine effectiveness, especially in specific groups such as elderly patients.

**Figure 4:**
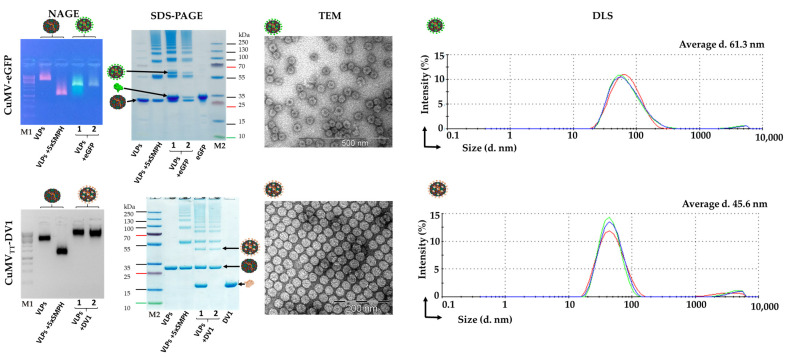
Integrity analysis of antigen coupling to VLPs. NAGE—coupling analysis in 0.8% native agarose gel electrophoresis, gel stained with ethidium bromide; bands indicate the presence of prokaryotic nucleic acid (mRNA) encapsulated inside the VLPs; SDS-PAGE—coupling analysis by 4–12% BoltPAGE stained with Coomassie G250, as a result of subunit crosslinking, derivatization by SMPH leads to the characteristic ladder of VLP monomers, dimers, trimers, tetramers, etc., indicating successful coupling reaction; TEM—analysis of VLPs after coupling by transmission electron microscopy at 80× (upper panel) or 100× (lower panel) magnifications; DLS—analysis of the uniformity and hydrodynamic diameter of VLPs after coupling by dynamic light scattering. Lanes in NAGE and SDS-PAGE: VLPs—uncoupled particles (negative control); VLPs + 5×SMPH—VLPs bound to the crosslinker (negative control); VLPs + eGFP/DV1: 1—VLPs loaded directly after coupling with antigen; 2—coupled VLPs loaded after removal of “free” (uncoupled) antigen; eGFP/DV1—“free” protein/peptide; M1—1 kb DNA ladder (GeneRuler^TM^ 1 kb, Thermo Fisher Scientific, Waltham, MA, USA, cat. SM0311); M2—protein molecular weight marker (PageRuler^TM^ Plus, Thermo Fisher Scientific, Waltham, MA, USA, cat. 26619).

The expression levels, purity, and efficiency of the conjugation process of eGFP to WT CuMV CP and DV1 to CuMV_TT_ CP were evaluated using 4–12% SDS-PAGE and 0.8% NAGE. The latter revealed successful conjugation of eGFP and DV1 to VLPs, as evidenced by the altered mobility of VLPs in the gel (Figure 4). Further confirmation of antigen-VLP conjugation quality was obtained through SDS-PAGE analysis, where a characteristic ladder could be observed, indicating a successful conjugation reaction (Figure 4). To assess the structural integrity and uniformity of the antigen-conjugated VLPs, TEM and DLS analyses were employed. TEM images demonstrated that the VLPs remained intact following the conjugation procedure. DLS analysis showed the presence of homogeneous peaks with average hydrodynamic diameters (D_h_) of about ~61 nm for CuMV-eGFP and ~46 nm for CuMV_TT_-DV1 (Figure 4). 

### 3.3. Characterization of the Immunogenic Potential of nCMV–eGFP Using “Immune-Tag” Technology

The efficacy of the immune response to a vaccine is significantly influenced by virus-like features. PAMPs and structural patterns such as size, geometry, and the presence of highly repetitive and ordered surface patterns (PASPs) play a crucial role in mimicking the natural attributes of viruses [3]. Some studies argue that simplified versions of VLPs, e.g., self-assembled viral nanostructures, may also serve as viral mimetics [208,209]. It can be hypothesized that incorporating additional immunostimulatory components into the “Immune-tag” nCMV-eGFP could increase Ab titers induced against the eGFP. This approach would facilitate the identification of the optimal configuration for future vaccine candidates utilizing the “Immune-tag”-based platforms. 

The immunogenicity of the developed eGFP-carrying nCMV “Immune-tag” constructs (Figure 1 and Figure 2) was evaluated through a vaccination regimen consisting of s.c. priming followed by two booster doses administered at 14-day intervals. To assess the impact of immunostimulatory elements on the immune response, we incorporated the PADRE element with the “Immune-tag” and/or supplemented it with ssRNA. For comparison, a “free” eGFP as well as CuMV-eGFP VLPs were used in the study (Figure 2). Serum samples from the mice were collected before the administration of the first dose and again at the endpoint of the study on Day 42. The total serum IgG levels as well as IgG1 and IgG2a subclasses against both eGFP and CuMV VLPs were measured by ELISA with the collected sera.

### 3.4. Total Levels of Anti-eGFP IgG

**Figure 5:**
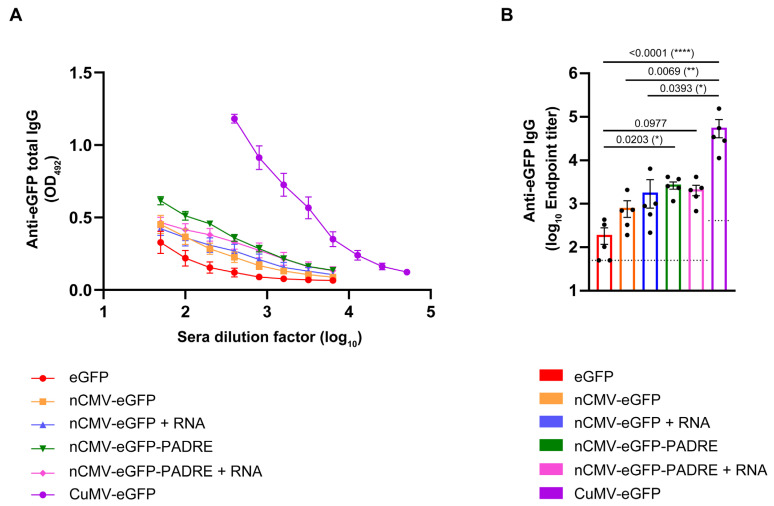
Analysis of eGFP-specific IgG titers following vaccination with vaccine variants. (**A**)—eGFP-specific IgG titers on day 42 for the groups vaccinated with nCMV-eGFP variants and CuMV-eGFP, measured at OD 492 nm; (**B**)—log_10_ of eGFP-specific IgG endpoint titers for the groups vaccinated with nCMV-eGFP variants and CuMV-eGFP. Statistical analysis (mean ± SEM) using the Kruskal-Wallis test. Vaccinated groups: *n* = 5. One representative experiment is shown. A value of *p* ≤ 0.05 was considered statistically significant (* *p* ≤ 0.05, ** *p* ≤ 0.01, *** *p* ≤ 0.001, **** *p* ≤ 0.0001).

There was a clear hierarchy in the eGFP-specific IgG responses. Groups of mice immunized with eGFP alone produced the lowest anti-eGFP IgG antibody titers, with mean reciprocal titers of 1:193 (Figure 5A,B). This result is consistent with the concept that oligomerization or multimerization of proteins into complexes can enhance immunogenicity by improving antigen recognition [210].

In comparison to eGFP alone, the experimental groups immunized with nCMV-based vaccines against eGFP exhibited a 4- to 14-fold increase in anti-eGFP IgG Ab titers (Figure 5A,B). The addition of each immunostimulatory element contributed to this rise in Ab titers. Interestingly, PADRE and RNA, when added individually, similarly boosted Ab responses, while the addition of both elements concurrently did not exhibit synergistic or even additive combined effects and resulted in a similar increase as the addition of each individual element separately. Importantly, the total eGFP-specific IgG titers in the group immunized with CuMV-eGFP VLPs were an order of magnitude higher than those measured in any other group, reaching mean reciprocal titers of 1:56,536 (Figure 5A,B). This suggests that the most effective immune responses are elicited when all viral components and features are integrated into a single entity.

CMV CP-specific IgG were detected exclusively in the sera of mice immunized with CuMV-eGFP (Figure S8). This observation is likely due to the nCMV fragment of CMV CP being concealed inside the particle.

### 3.5. Subclass-Specific Anti-eGFP IgG Antibodies

Literature underlines the importance of isotype class switching, IgG subclass differentiation, and the presence of high-affinity Abs as key factors influencing vaccine efficacy [211,212]. Specifically, IgG1 and IgG2a Ab subclasses serve as indicators of Th2 or Th1 responses, respectively. IgG2a rather than IgG1 engages activating Fc receptors, typically correlating with enhanced vaccine effectiveness [213,214]. The subclass-specific Ab response may strongly depend on TLR-stimulating factors such as RNA or DNA [215]. The preferential induction of IgG1 Abs was observed for influenza M2e domain-derived tetramers used for immunizations in BALB/c mice [216]. Conversely, Wang and co-workers [217] claimed that oligomers expressed by a DNA-based vaccine derived from the *Yersinia pestis* V antigen, which included a signal sequence from a human tissue plasminogen activator, elicited a dominant IgG2a response in the BALB/c murine model [217]. A similar observation was reported by Dalgediene and co-workers [218], where immunization of mice of the same strain with a polypeptide, the beta-amyloid (Aβ) oligomer, induced a predominantly IgG2a response [218]. These results indicate the importance of the origin of the oligomeric structures (e.g., viral, bacterial, fungal, mammalian, or self-created) in the stimulation of diverse IgG subclass responses, presumably via stimulation of different TLRs. To assess whether the developed “Immune-tags” could induce either IgG1 or IgG2a subclass-specific antibodies, blood sera from day 42 were analyzed.

**Figure 6:**
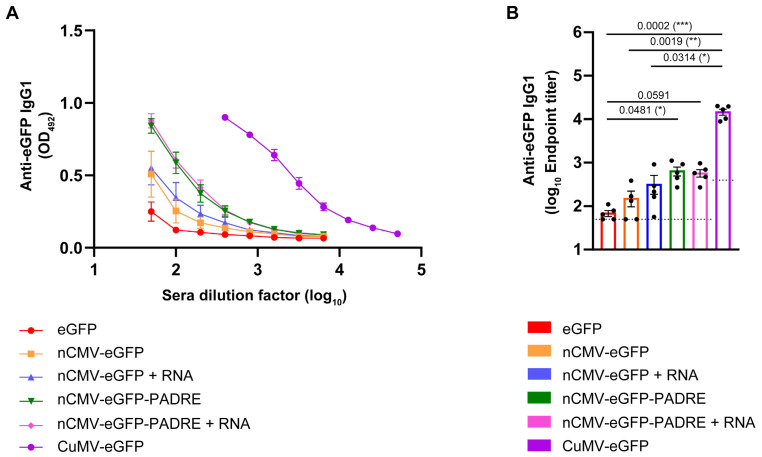
Induction of subclass switching to IgG1 by nCMV-eGFP variants and CuMV-eGFP determined by ELISA analysis. (**A**)—anti-eGFP-specific IgG1 titers measured on day 42 in mouse sera at OD 492 nm. (**B**)—log_10_ values of eGFP-specific IgG1 titers for the groups vaccinated with nCMV-eGFP variants and CuMV-eGFP. Statistical analysis (mean ± SEM) using the Kruskal-Wallis test. Vaccinated groups: *n* = 5. One representative experiment is shown. A value of *p* ≤ 0.05 was considered statistically significant (* *p* ≤ 0.05, ** *p* ≤ 0.01, *** *p* ≤ 0.001, **** *p* ≤ 0.0001).

Analysis of the Ab responses revealed a predominance of anti-eGFP IgG1 Abs across all tested groups. The highest response was observed in the group of mice immunized with chemically coupled CuMV-eGFP VLPs, achieving mean reciprocal titers of 1:15,131 (Figure 6A,B). When comparing nCMV-based “Immune-tags” to eGFP alone, a similar pattern of a 2- to 9-fold increase in eGFP-specific IgG1 Ab titers was observed, consistent with the measurements of total anti-eGFP IgG. This indicates that the majority of the detected total IgG was indeed of the IgG1 subclass. These findings align with a previously reported study, which identified IgG1 as the most prevalent Ab subclass in response to soluble protein antigens in mice [219,220,221,222].

eGFP-specific IgG2a Abs were detected only in the sera of mice vaccinated with CuMV-eGFP, reaching mean reciprocal titers of 1:2020 (Figure S9). These results suggest that constructed “Immune-tags” lack components that stimulate IgG2a production, in contrast to vaccines derived from whole VLPs. This aligns with previous findings that viruses induce IgG2a response in mice [221,222] and suggests that CP structural elements, when presented as multimerized “Immune-tags”, may not be recognized by the immune system in the same manner as the whole particle containing encapsulated nucleic acid.

### 3.6. nCMV-PADRE “Immune-Tag” as a Dengue Vaccine Candidate

To answer the question of whether the nCMV-PADRE “Immune-tag” may serve as an alternative platform for vaccine development, the immunogenicity of this construct was verified as a model anti-viral vaccine. For this purpose, the nCMV-PADRE was genetically fused with the DV1 EDIII (nCMV-PADRE-DV1; Figure 1 and Figure 2; Section 3.1). The Ig-like C-terminal EDIII of the DENV is known to be involved in the association of a virion with a receptor expressed on the surface of a host cell [223], serving as a crucial structure in viral entry. Consequently, EDIII contains epitopes that are exposed on the surface of the virion, constituting natural targets for the generation of neutralizing Abs [223,224].

**Figure 7:**
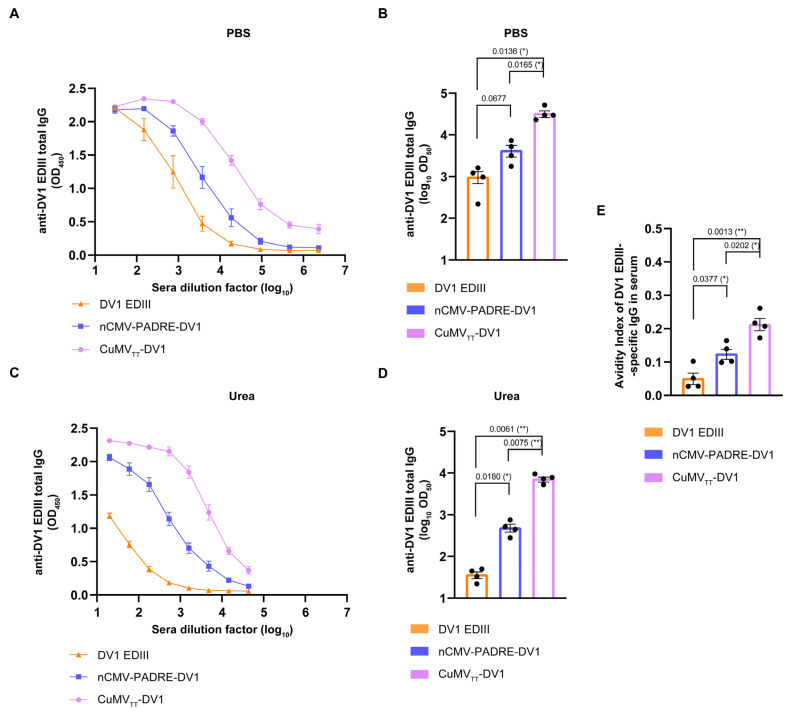
Anti-DV1 EDIII total IgG, high-avidity IgG titers after urea treatment, and avidity index (AI) calculation after vaccination with 30 µg of vaccine variants nCMV-PADRE-DV1, CuMV_TT_-DV1, and DV1 EDIII. (**A**)—Specific IgG titers against DV1 EDIII on Day 35 measured at OD 450 nm; (**B**)—log_10_ OD_50_ values of DV1 EDIII-specific IgG titers shown in A; (**C**)—DV1 EDIII-specific IgG titers on day 35, after three additional washes with 7 M urea, measured at OD 450 nm; (**D**)—log_10_ OD_50_ values of DV1 EDIII-specific IgG titers shown in C; (**E**)—avidity index of DV1 EDIII-specific serum IgG titers shown in B and D. Statistical analysis (mean ± SEM) was conducted using Brown-Forsythe and Welch ANOVA tests. Vaccinated groups, *n* = 4. One representative experiment is shown. A value of *p* ≤ 0.05 was considered statistically significant (* *p* ≤ 0.05, ** *p* ≤ 0.01, *** *p* ≤ 0.001, **** *p* ≤ 0.0001).

The immunogenicity of the newly constructed “Immune-tag” nCMV-PADRE-DV1 was evaluated in BALB/c mice. Animals were immunized s.c. with a 30 μg dose on Day 0, followed by a booster administered on Day 14. As negative and positive controls, the soluble DV1 EDIII and DV1 EDIII chemically coupled to the previously described “immunologically optimized” CuMV_TT_ (CuMV_TT_-DV1) [43] were used, respectively. The titers of serum total anti-DV1 EDIII IgG were measured at the endpoint of the experiment on Day 35 to assess the consistency of the IgG response hierarchy. Indeed, the IgG levels induced by the nCMV-PADRE-DV1 were higher than those in mice immunized with DV1 EDIII alone, confirming the relatively lower immunogenicity of the protein vaccines based on the recombinant DENV EDIII. Consistent with findings from the use of the eGFP model antigen, the anti-DV1 EDIII IgG levels were significantly highest in the group immunized with CuMV_TT_-DV1 (Figure 7A,B).

To compare the collective binding potency of the polyclonal serum anti-DV1 EDIII IgG Abs induced by CuMV_TT_-DV1, nCMV-PADRE-DV1, and DV1 EDIII alone, we performed an avidity ELISA assay. This assay was designed to include three intermediate 5-min plate washes with 7 M urea to reveal the differences between groups based on their specific IgG avidity. The use of this chaotropic agent eliminated low-affinity Abs from the samples (Figure 7C,D). The calculated AI revealed that nCMV-PADRE-DV1 induced significantly higher levels of high-affinity DV1 EDIII-specific IgG compared to “free” DV1 EDIII protein, while CuMV_TT_-DV1 induced the highest ratio of such IgGs of all vaccine candidates used (Figure 7E).

**Figure 8:**
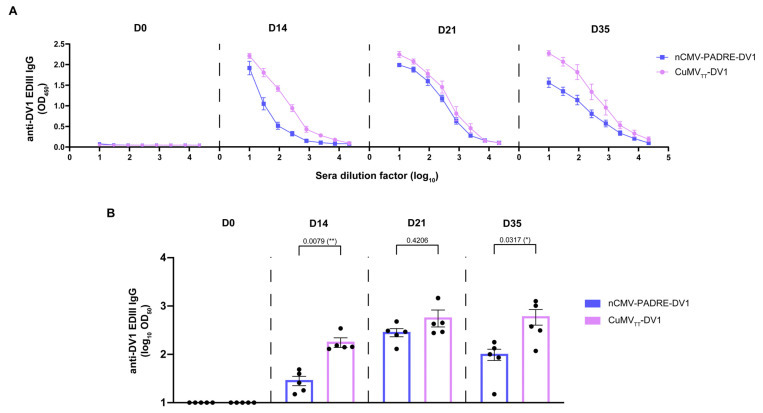
Analysis of anti-DV1 EDIII IgG titers over time following vaccination with nCMV-PADRE-DV1 and CuMV_TT_-DV1 vaccine variants. (**A**)—DV1 EDIII-specific IgG titers measured on days 0, 14, 21, and 35 at OD 450 nm; (**B**)—log_10_ OD_50_ values of DV1 EDIII-specific IgG titers shown in A. Statistical analysis (mean ± SEM) using Welch’s *t*-test. Vaccinated groups, *n* = 5. One representative experiment is shown. A value of *p* ≤ 0.05 was considered statistically significant (* *p* ≤ 0.05, ** *p* ≤ 0.01, *** *p* ≤ 0.001, **** *p* ≤ 0.0001).

Subsequently, BALB/c mice were immunized s.c. with a 10 μg dose of the “Immune-tag” nCMV-PADRE-DV1 or CuMV_TT_-DV1 following the same prime/boost regimen as shown before in Figure 7. Serum samples were collected before the prime on day 0, before the booster on day 14, as well as after the booster on days 21 and 35. A significantly higher levels of serum anti-DV1 EDIII IgG were observed on both day 14 and day 35 in the group of mice immunized with CuMV_TT_-DV1. Interestingly, after the boost, on day 21, the difference in levels of serum anti-DV1 EDIII IgG between the two groups was not significant (Figure 8A,B). This observation indicates that while CuMV_TT_-DV1 induced a stronger initial and longer-term IgG response against DV1 EDIII, during the immediate post-boost period there was no substantial difference in Ab levels between those two vaccine candidates.

**Figure 9:**
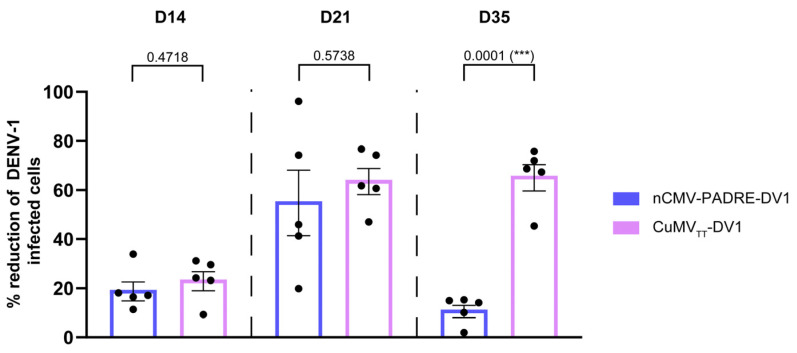
DENV-1 Focus Reduction Virus Neutralization Test (FRNT). FRNT shows the reduction of cells infected with DENV-1 incubated with sera from mice immunized with nCMV-PADRE-DV1 or CuMV_TT_-DV1 collected on days 14, 21, and 35 compared to sera collected on day 0. Statistical analysis (mean ± SEM) using Welch’s *t*-test. Vaccinated groups: *n* = 5. A value of *p* ≤ 0.05 was considered statistically significant (* *p* ≤ 0.05, ** *p* ≤ 0.01, *** *p* ≤ 0.001, **** *p* ≤ 0.0001).

Serum samples from mice immunized with 10 μg of oligomeric nCMV-PADRE-DV1 and CuMV_TT_-DV1 VLPs collected on Days 14, 21, and 35 were analyzed using a DENV-1 FRNT to assess the neutralizing capability of the Abs elicited by both vaccine constructs. The effectiveness of these Abs in neutralizing DENV-1 was determined by measuring the reduction in the number of virus-infected Vero cells in vitro, compared to a baseline established using cells infected by the virus incubated with pre-immune serum (collected on day 0). The results demonstrated that both vaccines were capable of inducing Abs that neutralized DENV-1 following the booster dose administered on day 21, as shown in Figure 9. However, the response induced by the oligomeric nCMV-PADRE-DV1 was found to be very transient, whereas the response from the VLP-based CuMV_TT_-DV1 vaccine was significantly more sustained. By day 35, the neutralizing capability of Abs induced by CuMV_TT_-DV1 was significantly greater than that induced by nCMV-PADRE-DV1, indicating a more durable protective effect with the VLP-based vaccine (Figure 9).

## 4. Discussion

Several structural elements derived from plant virus CPs were tested and used for multimeric structure formation as “artificial viral nanostructures”. By way of example, a 12-mer β-annulus peptide of Sesbania mosaic virus CP with FKFE sequence at the C-terminus self-assembles into a nanosphere of approximately 30 nm in diameter [79], and a 24-mer β-annulus peptide derived from the tomato bushy stunt virus CP spontaneously forms hollow “artificial viral capsids”, which can encapsulate anionic dyes, DNAs, quantum dots, and proteins and can be decorated with recombinant HRP or with a C-terminal His-tag [225]. Beyond plant viruses, peptides from other sources also demonstrated the capacity to self-assemble into nanostructures, serving as versatile tools for designing functional, supramolecular materials that are modular, tunable, and responsive to chemical and physical stimuli [84,209]. 

The N-terminal fragments of the R domains of CMV CPs form a bundle of six amphipathic helices [166]. This multimerization property could be used as an alternative vaccine platform—the “Immune-tag” [167]. To investigate the utility of those arginine-rich N-terminal domains (nCMVs), two constructs incorporating a model antigen eGFP, one including the additionally fused universal T-cell epitope PADRE [131,140], were initially developed. The use of eGFP was previously tested in the *Brucella abortus* S19 prototype vaccine as an associated diagnostic test to distinguish vaccinated animals from naturally infected with *Brucella* [226]. The incorporation of a universal T-cell epitope was intended to augment immune responses by inducing a strong Th-response against this epitope, which in turn would increase B-cell help [59]. Distinct versions of constructed “Immune-tags” were additionally supplemented with ssRNA to facilitate the formation of a six-helix bundle according to the CMV 3D structure [166] and serve as TLR7/8 stimulators.

Mouse immunization experiments showed a clear trend: the inclusion of additional immunostimulants consistently resulted in higher systemic IgG titers. Notably, the serum anti-eGFP IgG levels elicited by CuMV-eGFP VLPs were substantially higher than those triggered by the nCMV-PADRE-eGFP “Immune-tag”, regardless of RNA addition. These findings highlight the importance of a multifaceted approach in vaccine design, demonstrating that optimal immunogenicity relies not only on the inclusion of B-cell [227] and T-cell stimulating epitopes [43,59,151], or TLR ligands such as TLR3 [160,228] and TLR7/8 [160,229,230], but also on the physical properties of the particle/antigen delivery system. Specifically, the size of the particle [5,231,232,233], its multimerization [1,234], and its repetitiveness [114] play crucial roles in enhancing the immune response.

Building on the results of immunizations with eGFP-carrying “Immune-tags”, a dengue vaccine candidate, nCMV-PADRE-DV1, was developed. For this vaccine, DV1 EDIII, containing neutralizing epitopes, was selected as the experimental antigen. The humoral responses in the form of anti-DV1 EDIII IgG were significantly higher in sera collected from mice immunized with a VLP-based vaccine than in those immunized with a nCMV-based linear, multimerized construct. In addition, CuMV_TT_-DV1, following the boost, induced a prolonged and stable neutralizing response while such a response, induced by the oligomeric nCMV-PADRE-DV1 was transient only. The induction of high levels of neutralizing Abs against DENV-1 directly confirms that DV1 EDIII antigen maintains its authentic tertiary and quaternary structures. Given the well-established correlation between neutralizing Ab titers and protection in both humans and mice [235,236,237], our experimental data confirm the functional efficacy of our vaccine designs, even in the absence of direct DENV challenge data.

The neutralization ratio of DENV-1 can be attributed to several factors that may relate to both the quantity and quality of the elicited Abs. Our observations from the avidity ELISA assay indicate a significant increase in the ratio of high-affinity DV1 EDIII-specific IgG induced by nCMV-PADRE-DV1 compared to “free” DV1 EDIII protein. This underscores the effectiveness of incorporating the described stimulatory elements in enhancing the immunogenicity of the antigen. Importantly, we observed the ability of CuMV_TT_-DV1 to induce the highest ratio of high-affinity DV1 EDIII-specific IgG Abs among all tested vaccine constructs. Enhanced neutralization of DENV-1 by CuMV_TT_-DV1-induced serum post-boost contrasted with low neutralization despite relatively high DV1 EDIII-specific IgG responses observed pre-boost. This might be attributed to the initial Ab repertoire being predominantly composed of low-affinity Abs, which are less effective at DENV-1 neutralization [238,239]. This suggests that not only the quantity but also the quality of the Ab response may be critical for effective neutralization and long-term protection against DENV [240,241].

VLPs closely mimic the natural structure of viruses and present antigens in a repetitive, high-density manner that is optimal for B-cell receptor cross-linking and activation [1,2,3]. VLPs [242,243] and nanoparticle formulations have also been shown to be efficiently endocytosed by dendritic cells, which, in turn, leads to improved T-cell priming [244]. The design of VLPs/nanoparticles may promote a more balanced Th1/Th2 cell response [242,244], which leads to balanced Ab subclass responses [55,56,59,164]. In the context of generating the dengue vaccine candidates, the versatility of the VLP platforms allows for the exploration of VLPs derived directly from DENV (DENV VLPs) [180,245,246,247] or the use of other viral CP-based VLPs from sources such as mammalian viruses [182,248], plant viruses [50,51,249], or bacteriophages [77,250,251]. Broad comparative studies across different platforms could provide insight into their respective strengths and limitations in vaccine development, anti-dengue efficacy and safety. Meanwhile, our current study evaluates the impact of immunological cues and nanostructure patterns on the immune response, using the EDIII of DENV-1 as a biologically relevant model antigen.

Insights concerning targeting TLR4 and TLR3 with adjuvants (such as AS04 or NexaVant™, respectively) have been well documented in the literature [252,253]. This study introduces a novel perspective by specifically exploring the potential of targeting TLR7/8 within the framework of VLP-based vaccines. However, the mechanisms by which RNA influences immunogenicity are not yet fully understood. Our recently published study highlights the significant contribution of prokaryotic RNA encapsulated by the VLPs to the VLP-based vaccine’s immunogenicity through direct TLR7 and TLR3 engagement in B cells [160,254,255]. Here, we have not observed such a significant effect after supplementation of the “Immune-tags” with exogenous RNA. Moreover, the IgG responses induced by nCMV-based constructs (predominantly IgG1) resemble those triggered by proteins, and anti-viral protein-based vaccines often formulated with aluminum-based adjuvants [256,257]. Several factors may account for the observed differences in immunogenicity between the “Immune-tag”- and VLP-based vaccines: (1) the lack of sufficient doses/booster injections to potentially compensate for deficiencies in TLR 7 signaling and adequately increase the overall quantity of produced IgG Abs [160,255]; (2) the absence of nucleoside modifications characteristic of prokaryotic mRNA [125] within WT CMV CP mRNA obtained by in vitro transcription and supplemented to nCMV, what could impact the immune recognition [258,259,260]; (3) the insufficient amount of ssRNA [160], particularly lacking in polyU sequences [261,262], which are crucial for effective TLR7 stimulation; (4) suboptimal antigen distribution on the “Immune-tag” surface [116,160], potentially affecting the efficacy of antigen presentation; (5) the lack of dsRNA sequences that stimulate TLR3 [160,263]; (6) RNA degradation by RNases [264,265]; (7) reduced stability of the RNA during storage [266,267,268], which may be different if packaged within VLPs versus attached in the “free form” to multimers. Despite these challenges, the stimulation of immune responses by RNA needs further investigation, as achieving long-lasting immune responses is crucial for vaccinology. Recent experiences with COVID-19 highlight the complexity of designing effective vaccines [269].

When antigens are genetically fused to VLPs, the vaccine development process requires only single expression and purification steps. However, not all antigens can be effectively incorporated into the VLP structure through genetic fusion due to steric and/or folding issues. On the other hand, chemical conjugation requires a multistep process involving the separate expression and purification of both the VLP and antigen, followed by their conjugation and the final purification of the conjugated product. This prompts the search for alternative vaccine platforms and lead us to develop nCMV-based “Immune-tag” constructs. Such versatile antigen carriers overcome the limitations of direct genetic fusion methods, offering a potentially more cost-effective approach to vaccine design. The fact that the “Immune-tags” do not elicit Abs against the CMV CP provides an opportunity for their application as a booster vaccine after the administration of a first dose of CuMV_TT_ VLP-based vaccine [60,62,63,162,270,271,272], possibly with a suitable adjuvant. This feature suggests that the “Immune-tag”-based vaccines could be used to enhance and extend the immune response generated by the initial vaccination without the risk of cross-reactivity or interference with the primary vaccine’s antigenic targets [273,274,275,276]. The strategy of using “Immune-tag”-based vaccines has the potential to improve vaccination regimens but may also lead to increased cost efficiency in vaccine production [43,54,55,56,59]. This is particularly relevant when the production of a primary vaccine involves the chemical conjugation of an antigen to a carrier. The “Immune-tag” constructs can be expressed in *E. coli* culture in a single procedure with the same approach as VLPs. The capacity and flexibility of “Immune-tags” allow for the genetic fusion of multiple antigens and the incorporation of various effective immunostimulants while maintaining a simplified vaccine manufacturing process. Hence, VLP/”Immune-tag” combinations may be ideal for use in prime-boost regimens.

## Figures and Tables

**Table 1:** Nucleotides used for construct development.

Oligonucleotide Name	Sequence
CmN-NcoF	5′ ATACCATGGACAAATCTGAATCAACCAGT 3′
CmN-BamR	5′ TCTGGATCCCCGGTTGGGTGGTTAATAGTTGGACGA 3′
His-tag-C-eGFP-Bsp1407I-F	5′ AGCTGTACAAGGGTGGCGGATCCCATCATCATCATCATCACCATT 3′
His-tag-C-eGFP-SacI-R	5′ AGCGAGCTCTAGGGCCGCTTTAATGGTGATGATGATGATGATGGG 3′
pET-dir	5′ GGGGAATTGTGAGCGGATAACA 3′
pET-rev	5′ TATTGCTCAGCGGTGGCAGC 3’
M13seq-F	5’ GCCAGGGTTTTCCCAGTCACGA 3’
M13seq-R	5’ GAGCGGATAACAATTTCACACAGG 3’
PADRE-eGFP-BamHI-F	5′ ACCACCCAACCGGGGATCCCGCGAAATTTGTGGCCGCGTGGACCCTC 3’
PADRE-eGFP-AgeI-R	5′ TCACCATGGTGGCCACCGGTGGCGCGGCCGCCTTGAGGGTCCACGCGGCCAC 3’
nCMV-Vect_R	5′ TGCTCGAGAATTCAAGCTTGCTTTACAATAGCGGTGGCGCGGCCGCCT 3′

## Data Availability

The data supporting the findings of this study are available within the article and supporting materials. The data presented in this study are available upon request.

## Supplementary Materials

The following supporting information can be downloaded at: https://www.mdpi.com/article/10.3390/vaccines12060661/s1, Figure S1: nCMV-eGFP; Figure S2: nCMV-PADRE-eGFP; Figure S3: nCMV-PADRE-DV1; Figure S4: eGFP; Figure S5: DV1 EDIII; Figure S6: nCMV binding to the nucleic acid; Figure S7: Gel shift assays of nCMV-eGFP with distinct nucleic acid species; Figure S8: Analysis of anti-CuMV total IgG titers after vaccination with different vaccine variants; Figure S9: Analysis of anti-eGFP IgG2a subclass titers after vaccination with different vaccine variants.

## Abbreviations

**2,5-DHAP**	2,5-dihydroxyacetophenone
**Ab/Abs**	antibody/antibodies
**AEC**	addition of 3-amino-9-ethylcarbazole
**APCs**	antigen-presenting cells
**AI**	avidity index
**CMV**	cucumber mosaic virus
**CP**	capsid or coat protein
**CuMV**	cucumber mosaic virus-derived virus-like particles
**CuMV_TT_**	immunologically optimized cucumber mosaic virus-derived VLPs with incorporated universal Th-cell epitope from tetanus toxin
**DENV**	dengue virus
**DLS**	dynamic light scattering
**DV1 EDIII**	third domain of dengue virus 1 envelope protein
**eGFP**	enhanced green fluorescent protein
**ELISA**	enzyme-linked immunosorbent assay
**FRNT**	focus reduction virus neutralization test
**IB**	inclusion bodies
**MS**	mass spectrometry
**NAGE**	native agarose gel electrophoresis
**nCMV**	N-terminal fragment of the cucumber mosaic virus capsid protein containing the functional R domain
**ON**	overnight
**PADRE**	universal synthetic non-natural Pan DR Epitope
**PAMPs**	pathogen-associated molecular patterns
**PASPs**	Pathogen-associated structural patterns
**PMSF**	phenylmethylsulfonyl fluoride
**RT**	room temperature
**SATA**	N-succinimidyl S-acetylthioacetate
**s.c.**	subcutaneous
**SDS-PAGE**	sodium dodecyl sulfate-polyacrylamide gel electrophoresis
**SMPH**	succinimidyl 6-((β-maleimidopropionamido) hexanoate
**TEM**	transmission electron microscopy
**Th**	T-cell help
**Th cell**	T-helper cell
**TLR**	toll-like receptor
**TMB**	tetramethylbenzidine
**TT**	tetanus toxin
**VLPs**	virus-like particles
**WT**	wild type
