# Supplementary material for: Identifying Key Drivers of Efficient B Cell Responses: On the Role of T Help, Antigen-Organization, and Toll-like Receptor Stimulation for Generating a Neutralizing Anti-Dengue Virus Response
Source: Vaccines (Basel). 2024 Jun 14;12(6):661. doi: 10.3390/vaccines12060661 (PMC11209419; doi:10.3390/vaccines12060661)
Supplement: Supplementary file 1 [file vaccines-12-00661-s001.zip › vaccines-2945232-supplementary.pdf]

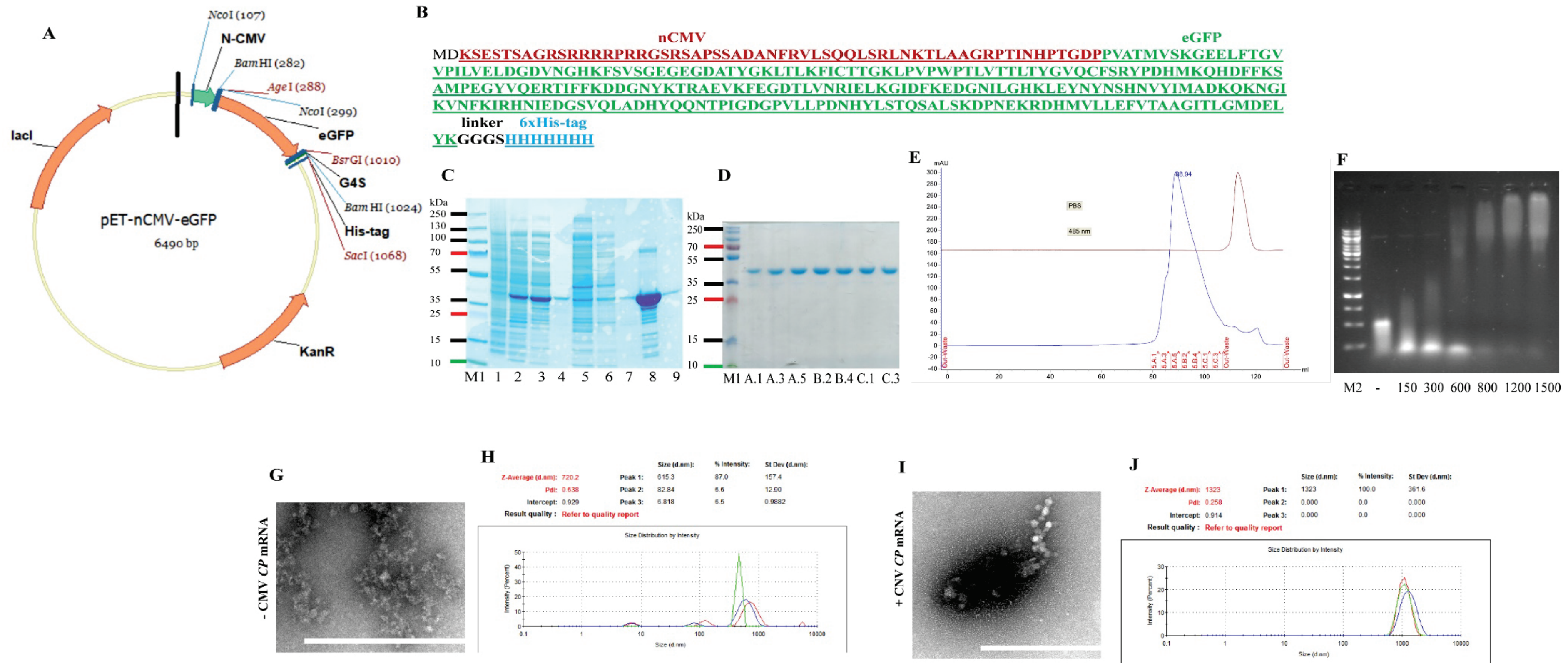

Figure S1. nCMV-eGFP. (**A**) – expression plasmid scheme; (**B**) – amino acid sequence of nCMV-eGFP "Immune-tag" elements; (**C**) – SDS-PAGE analysis of nCMV-eGFP solubility and its purification using a His-tag column; stained with Coomassie G250; (**D**) – SDS-PAGE analysis of nCMV-eGFP gel-filtration fractions; stained with Coomassie G250; (**E**) – gel-filtration chromatography chromatogram; (**F**) – gel shift analysis of nCMV-eGFP supplemented with CMV *CP* mRNA in 1% native agarose gel stained with ethidium bromide; (**G**) – analysis of nCMV-eGFP without supplemented CMV *CP* mRNA by transmission electron microscopy (TEM) in 100× resolution (white bar represents 500 nm scale); (**H**) – analysis of nCMV-eGFP without supplemented CMV *CP* mRNA by dynamic light scattering (DLS); (**I**) – analysis of nCMV-eGFP with supplemented CMV *CP* mRNA by TEM in 100× resolution (white bar represents 500 nm scale); (**J**) – analysis of nCMV-eGFP with supplemented CMV *CP* mRNA by DLS; M1 – protein molecular weight marker (PageRuler™ Plus, Thermo Fisher Scientific, Waltham, MA, USA, cat. 26619); M2 – 1 kb DNA ladder (GeneRuler™ 1 kb, Thermo Fisher Scientific, Waltham, MA, USA, cat. SM0311); 1 – total cell lysate before cell induction; 2 – total cell lysate after cell induction; 3 – soluble protein fraction; 4 – insoluble protein fraction; 5 – sample from flow-through fraction of His-tag column; 6,7 – sample from wash fractions of His-tag column; 8,9 – elution fractions from His-tag column; A.1-C.3 – gel-filtration fractions; «-» – CMV *CP* mRNA; 150 – CMV *CP* mRNA with 150 ng protein; 300 – CMV *CP* mRNA with 300 ng protein, 600 – CMV *CP* mRNA with 600 ng protein, 800 – CMV *CP* mRNA with 800 ng protein, 1200 – CMV *CP* mRNA with 1200 ng protein, 1500 – CMV *CP* mRNA with 1500 ng protein.

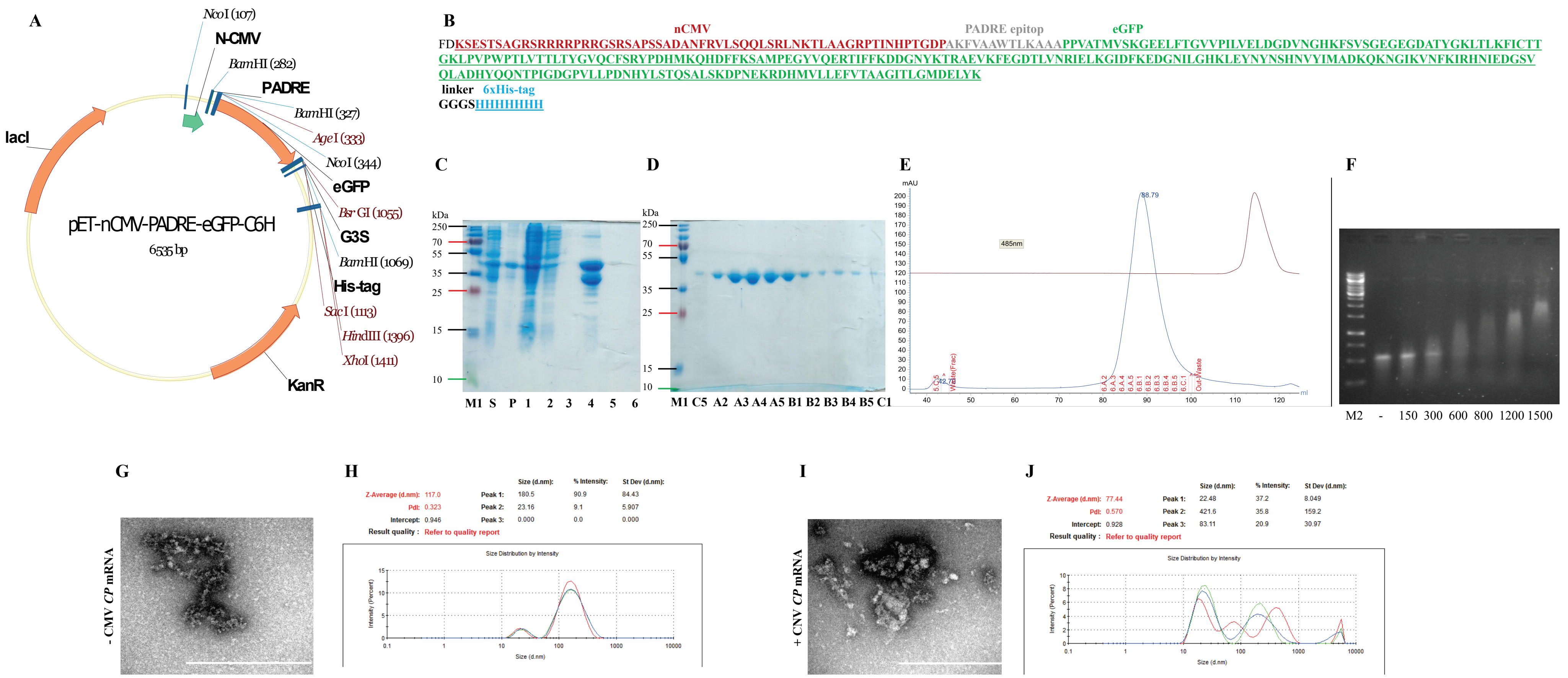

Figure S2. nCMV-PADRE-eGFP. (**A**) – expression plasmid scheme; (**B**) – amino acid sequence of nCMV-PADRE-eGFP “Immune-tag” elements; (**C**) SDS-PAGE analysis of nCMV-PADRE-eGFP solubility and its purification using a His-tag column; stained with Coomassie G250; (**D**) – SDS-PAGE analysis of nCMV-PADRE-eGFP gel-filtration fractions; stained with Coomassie G250; (**E**) – gel-filtration chromatography chromatogram; (**F**) – gel shift analysis of nCMV-PADRE-eGFP supplemented with CMV *CP* mRNA in 1% native agarose gel stained with ethidium bromide; (**G**) – analysis of nCMV-PADRE-eGFP without supplemented CMV *CP* mRNA by TEM in 100× resolution (white bar represents 500 nm scale); (**H**) – analysis of nCMV-PADRE-eGFP without supplemented CMV *CP* mRNA by DLS; (**I**) – analysis of nCMV-PADRE-eGFP with supplemented CMV *CP* mRNA by TEM in 100× resolution (white bar represents 500 nm scale); (**J**) – analysis of nCMV-PADRE-eGFP with supplemented CMV *CP* mRNA by DLS; M1 – protein molecular weight marker (PageRuler™ Plus, Thermo Fisher Scientific, Waltham, MA, USA, cat. 26619); M2 – 1 kb DNA ladder (GeneRuler™ 1 kb, Thermo Fisher Scientific, Waltham, MA, USA, cat. SM0311); S – soluble protein fraction; P – insoluble protein fraction; 1 – sample from flow-through fraction of His-tag column; 2,3 – sample from wash fractions of His-tag column; 4,5,6 – elution fractions from His-tag column; C5-C1 – gel-filtration fractions; «-» – CMV *CP* mRNA; 150 – CMV *CP* mRNA with 150 ng protein; 300 – CMV *CP* mRNA with 300 ng protein, 600 – CMV *CP* mRNA with 600 ng protein, 800 – CMV *CP* mRNA with 800 ng protein, 1200 – CMV *CP* mRNA with 1200 ng protein, 1500 – CMV *CP* mRNA with 1500 ng protein.

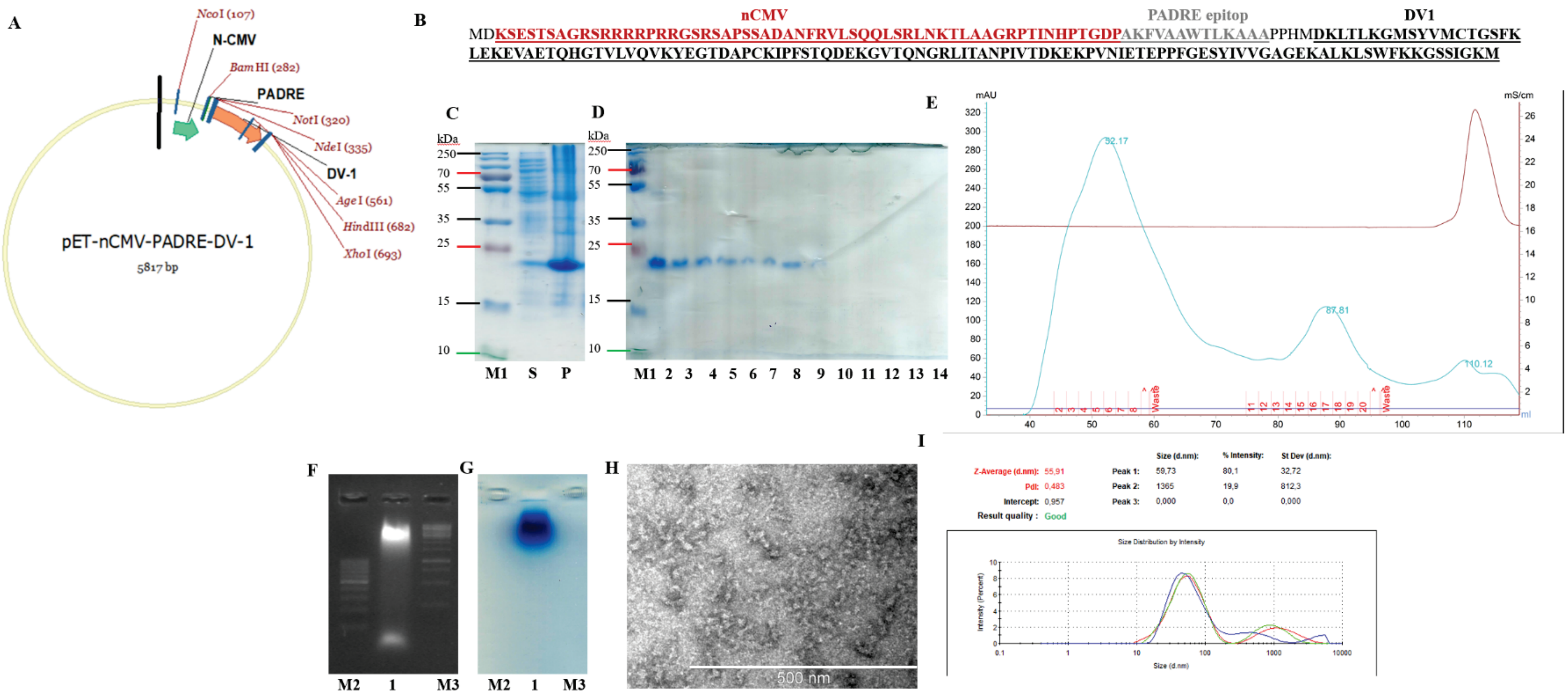

Figure S3. nCMV-PADRE-DV1. (**A**) – expression plasmid scheme; (**B**) – amino acid sequence of nCMV-PADRE-DV1 "Immune-tag" elements; (**C**) – SDS-PAGE analysis of nCMV-PADRE-DV1 solubility after cell disruption; stained with Coomassie G250; (**D**) – SDS-PAGE analysis of nCMV-PADRE-DV1 gel-filtration fractions; stained with Coomassie G250; (**E**) – gel-filtration chromatography chromatogram; (**F**) – analysis of nCMV-PADRE-DV1 supplemented with WT CMV *CP* mRNA on 0.8% native agarose gel stained with ethidium bromide; (**G**) – analysis of nCMV-PADRE-DV1 supplemented with WT CMV *CP* mRNA on 0.8% native agarose gel stained with Coomassie G250; (**H**) – analysis of nCMV-PADRE-DV1 supplemented with WT CMV *CP* mRNA by TEM, 100× resolution (white bar represents 500 nm scale); (**I**) – analysis of nCMV-PADRE-DV1 supplemented with WT CMV *CP* mRNA by DLS; M1 – protein molecular weight marker (PageRuler^{TM} Plus, Thermo Fisher Scientific, Waltham, MA, USA, cat. 26619); M2 – 1 kb DNA ladder (GeneRuler^{TM} 1 kb, Thermo Fisher Scientific, Waltham, MA, USA, cat. SM0311); M3 – 100 DNA bp ladder (GeneRuler^{TM} 100 bp Plus DNA Ladder, Thermo Fisher Scientific, Waltham, MA, USA, cat. SM0321); S – soluble protein fraction; P – insoluble protein fraction; 1 – purified nCMV-PADRE-DV1; 2-14 – gel-filtration fractions.

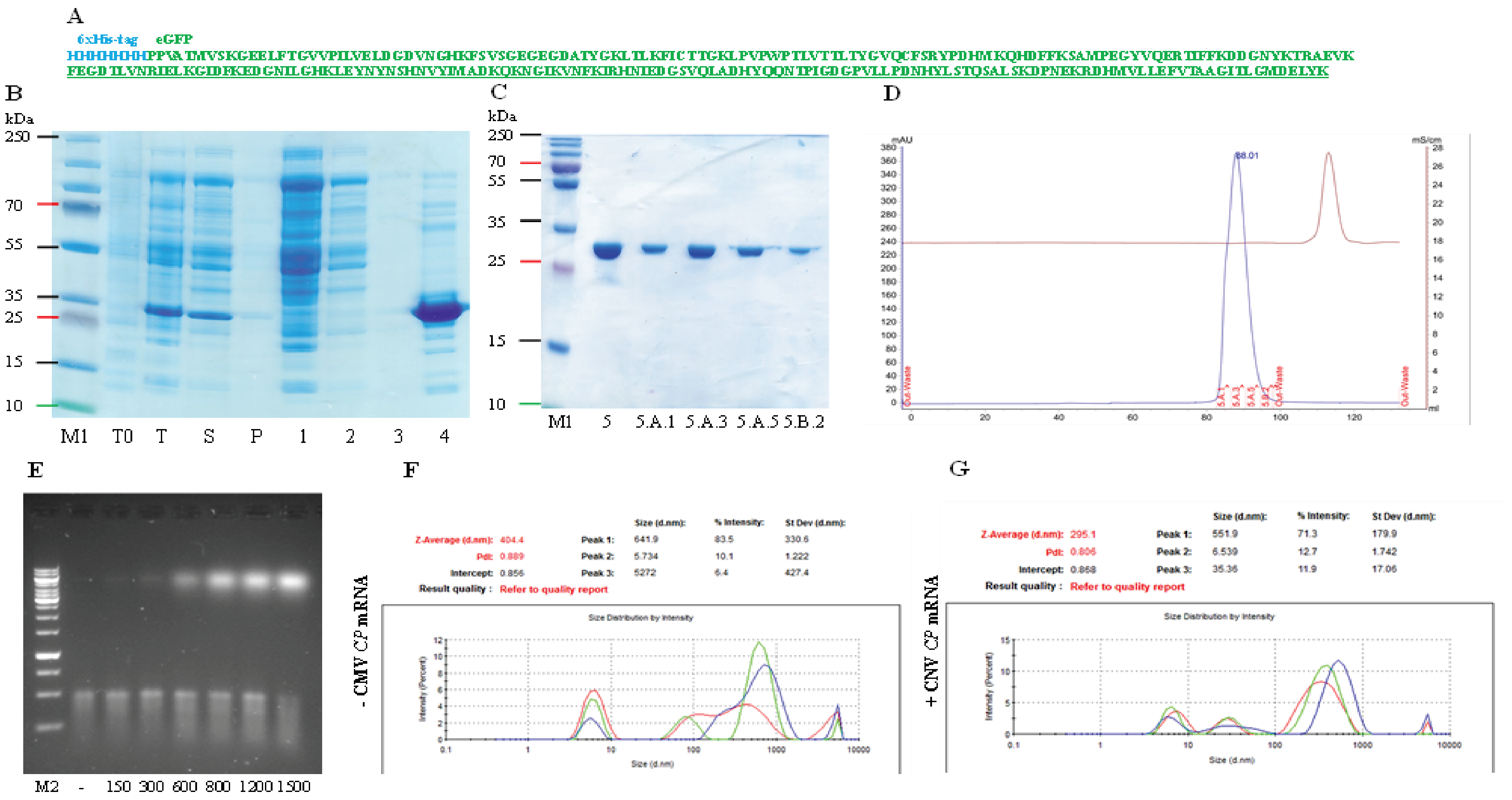

Figure S4. eGFP. (**A**) – eGFP amino acid sequence; (**B**) – SDS-PAGE analysis of eGFP solubility and its purification using a His-tag column; stained with Coomassie G250; (**C**) – SDS-PAGE analysis of eGFP gel-filtration fractions; stained with Coomassie G250; (**D**) – gel-filtration chromatography chromatogram; (**E**) – gel shift analysis of eGFP supplemented with CMV *CP* mRNA in 1% native agarose gel stained with ethidium bromide; (**F**) – analysis of eGFP without supplemented CMV *CP* mRNA by DLS; (**G**) – analysis of nCMV-PADRE-eGFP with supplemented CMV *CP* mRNA by dynamic DLS; M1 – protein molecular weight marker (PageRuler^{TM} Plus, Thermo Fisher Scientific, Waltham, MA, USA, cat. 26619); M2–1 kb DNA ladder (GeneRuler^{TM} 1 kb, Thermo Fisher Scientific, Waltham, MA, USA, cat. SM0311); T0 – total cell lysate before cell induction; T – total cell lysate after cell induction; S – soluble protein fraction; P – insoluble protein fraction; 1 – sample from flow-through fraction of His-tag column; 2,3 – samples from wash fractions of His-tag column; 4 –elution fraction from His-tag column; 5 – eGFP after concentration on Amicon$^{®}$ Ultra-15, 10 KDa MWCO filtration unit; 5.A1-5.B.2 – gel-filtration fractions; «-» – CMV *CP* mRNA; 150 – CMV *CP* mRNA with 150 ng protein; 300 – CMV *CP* mRNA with 300 ng protein, 600 – CMV *CP* mRNA with 600 ng protein, 800 – CMV *CP* mRNA with 800 ng protein, 1200 – CMV *CP* mRNA with 1200 ng protein, 1500 – CMV *CP* mRNA with 1500 ng protein.

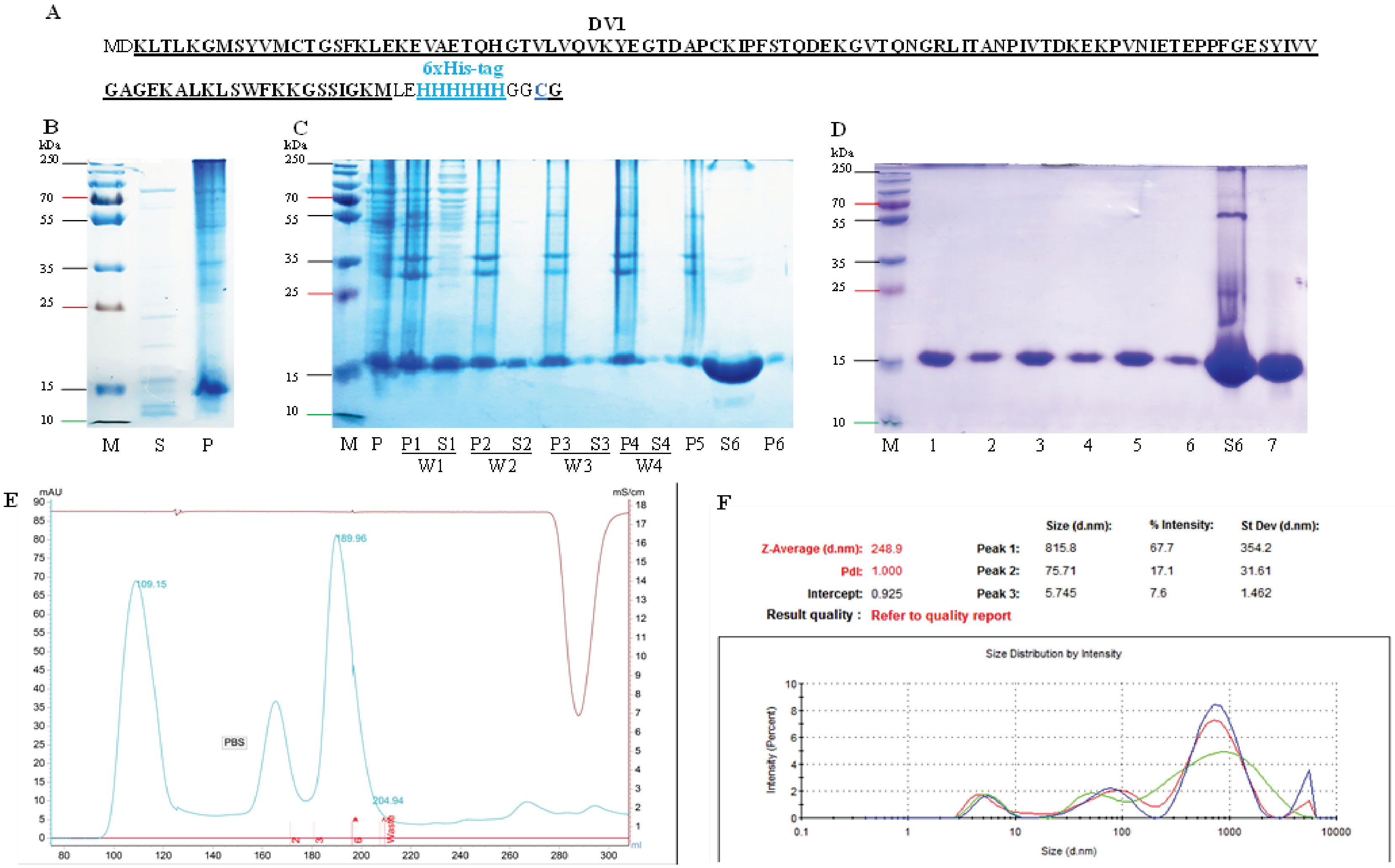

Figure S5. DV1 EDIII. (**A**) – DV1 EDIII amino acid sequence; (**B**) – SDS-PAGE analysis of DV1 EDIII solubility after cell disruption; stained with Coomassie G250; (**C**) – SDS-PAGE analysis of inclusion bodies (IB) wash steps and refolding; stained with Coomassie G250; (**D**) – SDS-PAGE analysis of DV1 EDIII gel-filtration fractions; stained with Coomassie G250; (**E**) – gel-filtration chromatography chromatogram; (**F**) – analysis of DV1 EDIII by DLS; M – protein molecular weight marker (PageRuler^{TM} Plus, Thermo Fisher Scientific, Waltham, MA, USA, cat. 26619); S – soluble protein fraction; P – insoluble protein fraction; W1 – IB analysis after first wash; W2 – IB analysis after second wash; W3 – IB analysis after third wash; W4 – IB analysis after fourth wash; 1-6 – gel-filtration fractions; 7 – DV1 EDIII after concentration with Amicon® Ultra-15, 10 KDa MWCO filtration unit.

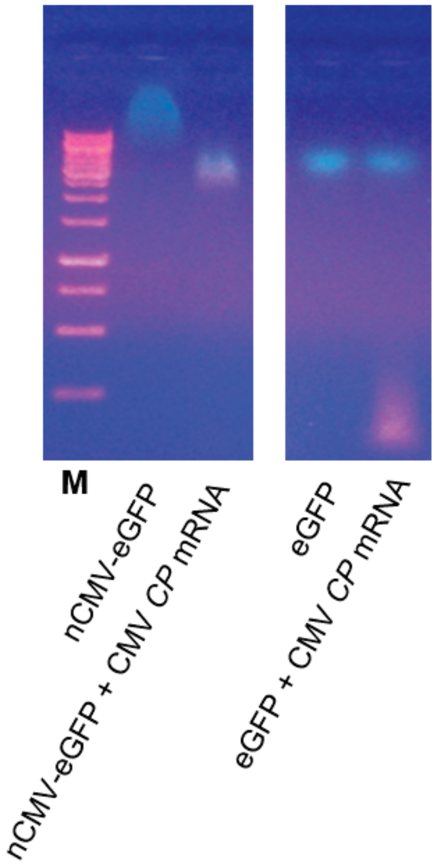

Figure S6. nCMV binding to the nucleic acid. 1 µg of eGFP (negative control), and nCMV-eGFP vaccine were supplemented with 1 µg of WT CMV *CP* mRNA and analyzed using 0.8% NAG stained with ethidium bromide. M – 1 kb DNA ladder (GeneRuler^{TM} 1 kb, Thermo Fisher Scientific, Waltham, MA, USA, cat. SM0311).

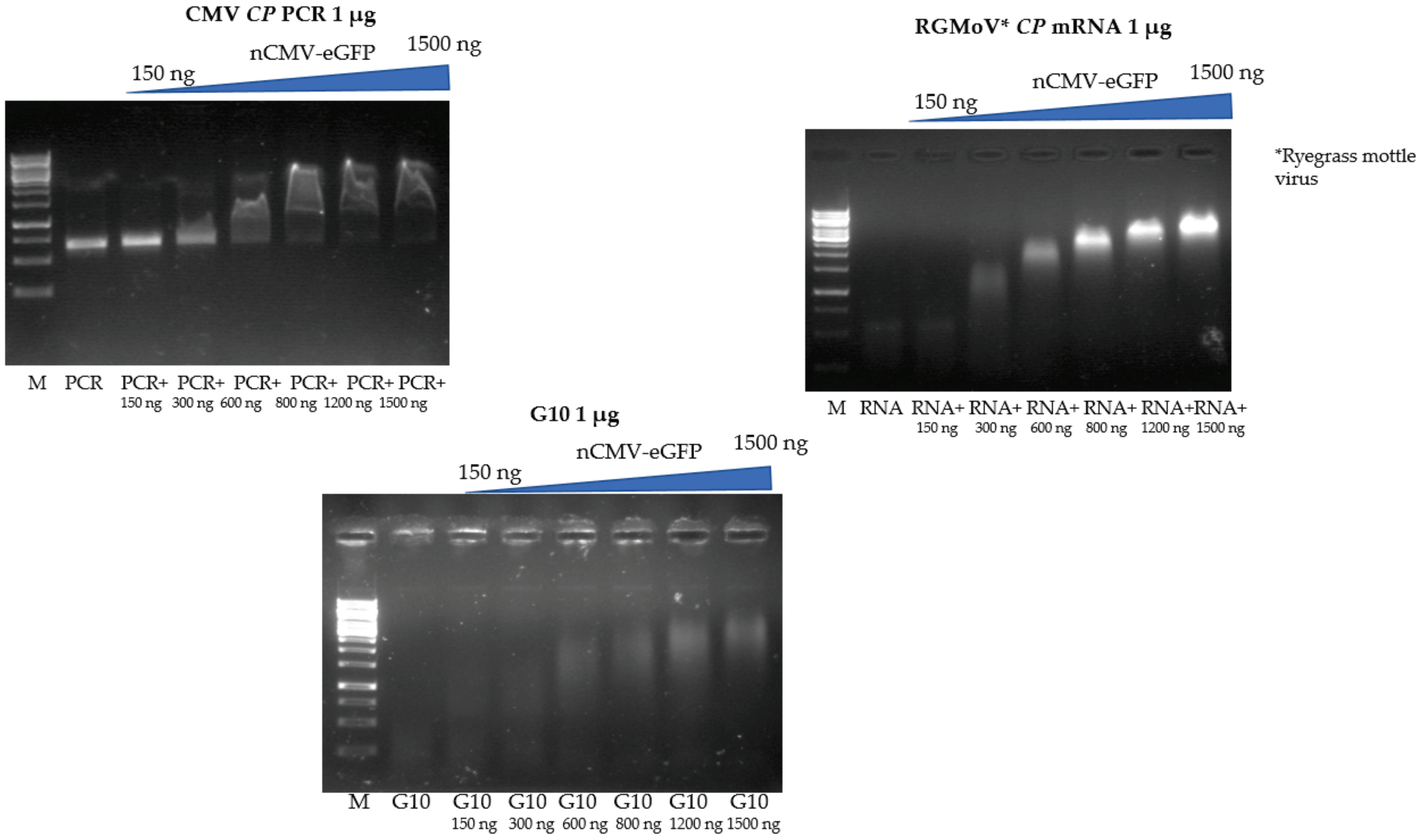

Figure S7. Gel shift assays of nCMV-eGFP with distinct nucleic acid species. The nCMV-eGFP vaccine variant was evaluated across seven doses (0, 150, 300, 600, 800, 1200, and 1500 ng) for its capacity to bind various types of nucleic acids: CMV *CP* PCR product, ryegrass mottle virus (RGMoV) *CP* mRNA, and the Type A CpG TLR9 agonist - G10. Analysis was performed on a 1% NAG gel stained with ethidium bromide. M – 1 kb DNA ladder (GeneRuler™ 1 kb, Thermo Fisher Scientific, Waltham, MA, USA, cat. SM0311).

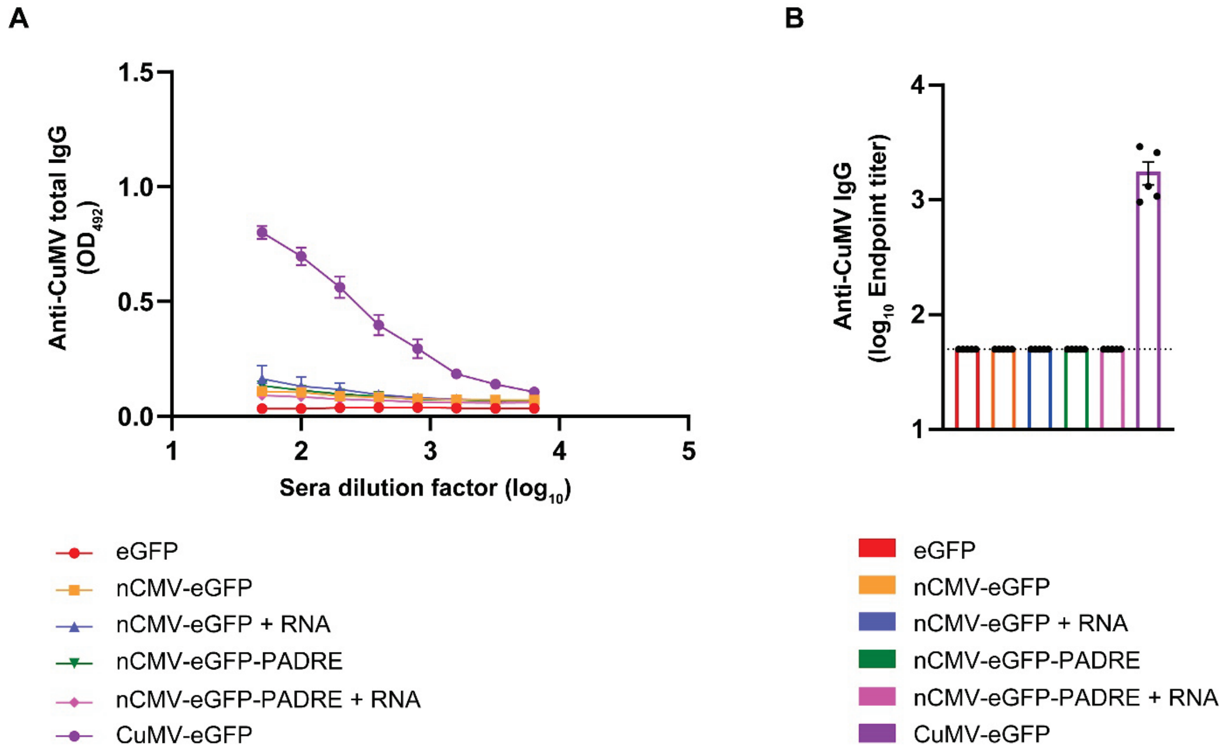

Figure S8. Analysis of anti-CuMV total IgG titers after vaccination with different vaccine variants. (**A**) – CuMV-specific IgG titers on Day 42 for the groups vaccinated with nCMV-eGFP variants and CuMV-eGFP measured at OD 492 nm; (**B**) – $\log_{10}$ values (mean ± SEM) of CuMV-specific IgG titers for the groups vaccinated with nCMV-eGFP variants and CuMV-eGFP.

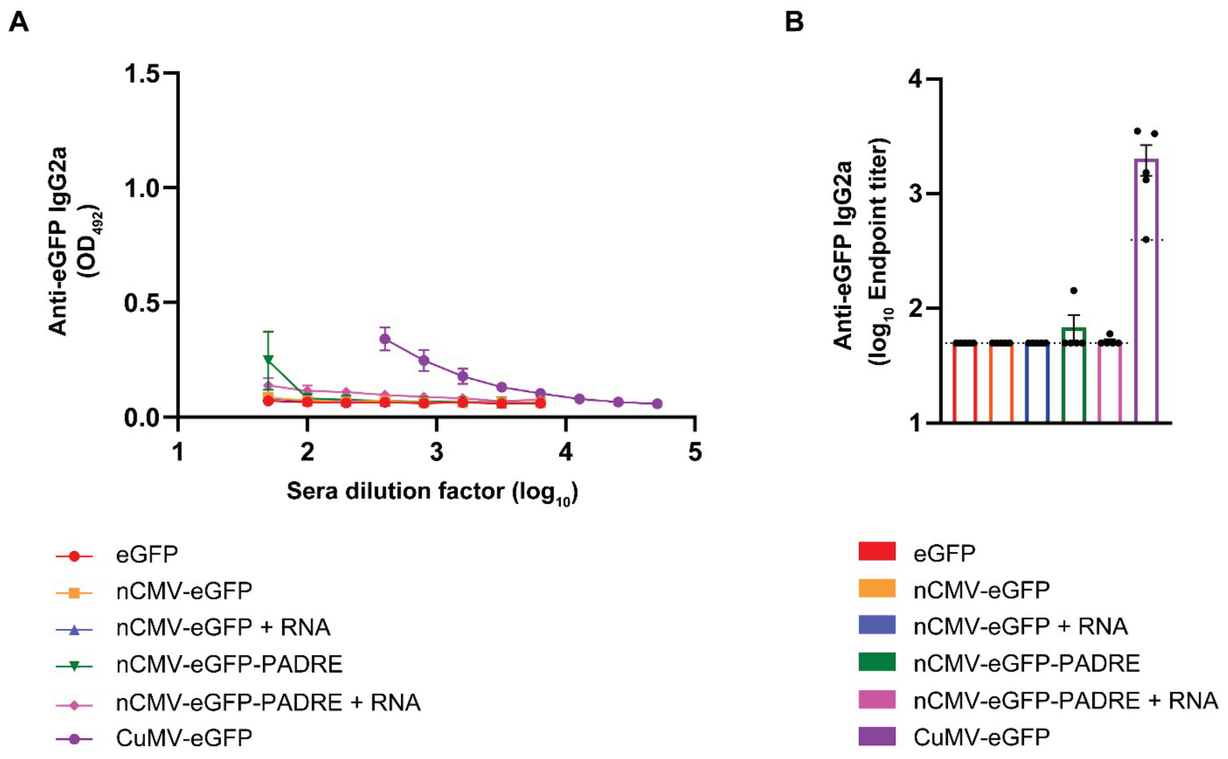

Figure S9. Analysis of anti-eGFP IgG2a subclass titers after vaccination with different vaccine variants. (**A**) – Anti–eGFP specific IgG2a titers measured in Day 42 mouse sera at OD492 nm. ELISA plates were coated with nCMV-eGFP variants and CuMV-eGFP to detect IgG subclasses in mice vaccinated with nCMV-eGFP variants and CuMV-eGFP. (**B**) – $\log_{10}$ values (mean ± SEM) of eGFP-specific IgG2a titers for the groups vaccinated with nCMV-eGFP variants and CuMV-eGFP.
